# Progress in Brain Computer Interface: Challenges and Opportunities

**DOI:** 10.3389/fnsys.2021.578875

**Published:** 2021-02-25

**Authors:** Simanto Saha, Khondaker A. Mamun, Khawza Ahmed, Raqibul Mostafa, Ganesh R. Naik, Sam Darvishi, Ahsan H. Khandoker, Mathias Baumert

**Affiliations:** ^1^School of Electrical and Electronic Engineering, The University of Adelaide, Adelaide, SA, Australia; ^2^Department of Electrical and Electronic Engineering, United International University, Dhaka, Bangladesh; ^3^Advanced Intelligent Multidisciplinary Systems (AIMS) Lab, Department of Computer Science and Engineering, United International University, Dhaka, Bangladesh; ^4^Adelaide Institute for Sleep Health, College of Medicine and Public Health, Flinders University, Adelaide, SA, Australia; ^5^Healthcare Engineering Innovation Center, Department of Biomedical Engineering, Khalifa University of Science and Technology, Abu Dhabi, United Arab Emirates

**Keywords:** brain computer interface, hybrid/multimodal BCI, neuroimaging techniques, neurosensors, electrical/hemodynamic brain signals, cognitive rehabilitation

## Abstract

Brain computer interfaces (BCI) provide a direct communication link between the brain and a computer or other external devices. They offer an extended degree of freedom either by strengthening or by substituting human peripheral working capacity and have potential applications in various fields such as rehabilitation, affective computing, robotics, gaming, and neuroscience. Significant research efforts on a global scale have delivered common platforms for technology standardization and help tackle highly complex and non-linear brain dynamics and related feature extraction and classification challenges. Time-variant psycho-neurophysiological fluctuations and their impact on brain signals impose another challenge for BCI researchers to transform the technology from laboratory experiments to plug-and-play daily life. This review summarizes state-of-the-art progress in the BCI field over the last decades and highlights critical challenges.

## 1. Introduction

**Figure 1:**
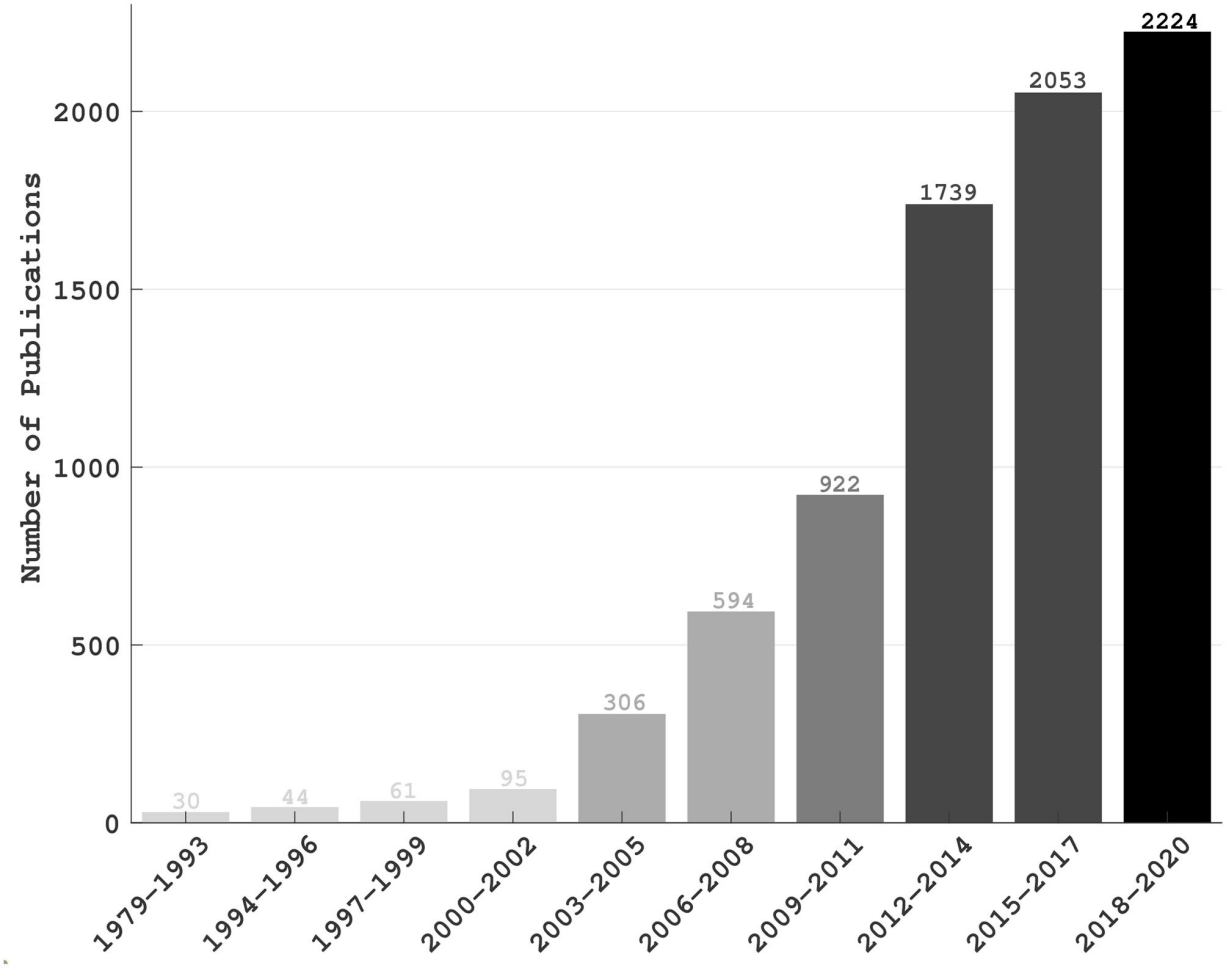
The number of publications over the years: The statistics was based on a search on PubMed in which “brain computer interface” was the search keyword. The publications those were listed until 4th December 2020 have been accounted only. A significant increase in the number of publications in this decade as compared to the last decade implicates the engagement of a greater community in this field and, thus the importance of BCI technology.

The brain computer interface (BCI) is a direct and sometimes bidirectional communication tie-up between the brain and a computer or an external device, which involves no muscular stimulation. It has shown promise for rehabilitating subjects with motor impairments as well as for augmenting human working capacity either physically or cognitively (Lebedev and Nicolelis, 2017; Saha and Baumert, 2020). BCI was historically envisioned as a potential technology for augmenting/replacing existing neural rehabilitations or serving assistive devices controlled directly by the brain (Vidal, 1973; Birbaumer et al., 1999; Alcaide-Aguirre et al., 2017; Shahriari et al., 2019). The first systematic attempt to implement an electroencephalogram (EEG)-based BCI was made by J. J. Vidal in 1973, who recorded the evoked electrical activity of the cerebral cortex from the intact skull using EEG (Vidal, 1973), a non-invasive technique first studied in humans invented by Berger (1929). Another early endeavor to establish direct communication between a computer and the brain of people with severe motor impairments had utilized P300, an event related brain potential (Farwell and Donchin, 1988). As an alternative to conventional therapeutic rehabilitation for motor impairments, BCI technology helps to artificially augment or re-excite synaptic plasticity in affected neural circuits. By exploiting undamaged cognitive and emotional functions, BCI aims at re-establishing the link between the brain and an impaired peripheral site (Vansteensel et al., 2016). However, the research applications of BCI technology evolved significantly over the years, including brain fingerprinting for lie detection (Farwell et al., 2014), detecting drowsiness for improving human working performances (Aricò et al., 2016; Wei et al., 2018), estimating reaction time (Wu et al., 2017b), controlling virtual reality (Vourvopoulos et al., 2019), quadcopters (LaFleur et al., 2013) and video games (Singh et al., 2020), and driving humanoid robots (Choi and Jo, 2013; Spataro et al., 2017). Figure 1 demonstrates the progression of BCI in various application fields since its conception.

According to the Brain/Neural Computer Interaction Horizon 2020 project, an initiative by the European Commission for coordinating BCI research, six major application themes, i.e., restore (e.g., unlocking the completely locked-in), replace (e.g., BCI-controlled neuroprosthesis), enhance (e.g., enhanced user experience in computer games), supplement (e.g., augmented reality glasses), improve (e.g., upper limb rehabilitation after stroke), and research tool (e.g., decoding brain activity with real-time feedback) have been outlined as feasible and promising fields (Brunner et al., 2015). This overview encompasses a wide range of challenges and trends in BCI field. For specialized reviews on particular BCI topics we refer to the recent literature (McFarland et al., 2006; Schwartz et al., 2006; Bashashati et al., 2007; Lotte et al., 2007, 2018; Matthews et al., 2007; Sitaram et al., 2007; Mak and Wolpaw, 2009; Menon et al., 2009; Nicolelis and Lebedev, 2009; Summerer et al., 2009; Vaadia and Birbaumer, 2009; Milan and Carmena, 2010; Min et al., 2010; Clausen, 2011; Krusienski et al., 2011; Liao et al., 2012; Nicolas-Alonso and Gomez-Gil, 2012; Shih et al., 2012; Jebari, 2013; McCullagh et al., 2014; Ahn and Jun, 2015; Jayaram et al., 2016; Lebedev and Nicolelis, 2017; Mudgal et al., 2020; Rashid et al., 2020; Saha and Baumert, 2020).

### 1.1. Characterization of BCI Systems

BCI systems can be categorized by the way they use the brain: Passive BCI decode unintentional affective/cognitive states of the brain (Zander et al., 2009), while active BCI directly involve the user's voluntary intention-induced brain activity. Reactive BCI use brain waves generated as response to external stimuli. Detecting driver's drowsiness to prevent road accidents is an example of passive BCI (Lin et al., 2008; Gao et al., 2019). BCI systems driven by users' intentional motor imagery (MI) (Marchesotti et al., 2016; Saha et al., 2019a; Saha and Baumert, 2020) and visually evoked P300 produced by external stimulation (Farwell et al., 2014) can be considered active BCI and reactive BCI, respectively.

The modality of signal acquisition has been used to divide systems into invasive and non-invasive BCI (Min et al., 2010; Rosenfeld and Wong, 2017). Non-invasive BCI exploiting EEG are most common, although more recently, functional near infrared spectroscopy (fNIRS) (Matthews et al., 2007), magnetoencephalography (MEG) (Fukuma et al., 2016), functional magnetic resonance imaging (fMRI) (Kaas et al., 2019) and functional transcranial Doppler ultrasonography (Faress and Chau, 2013; Lu et al., 2015; Khalaf et al., 2019) have been exploited. In contrast, invasive intracortical electrodes (Pandarinath et al., 2017) and electrocorticography (ECoG) (Kaiju et al., 2017) have been used, providing a superior signal-to-noise ratio and better localization of brain activity. Table 1 summarizes signal acquisition modalities and their suitability for BCI applications.

**Table 1:** A list of neuroimaging techniques and their suitability in brain computer interface (BCI) applications.

**Feature**	**EEG**	**MEG**	**ECoG**	**Intracortical Recording**	**fMRI**	**fNIRS**	**PET**
Activity type	Electrical	Magnetic	Electrical	Electrical	Metabolic	Metabolic	Metabolic
Measurement type	Direct	Direct	Direct	Direct	Indirect	Indirect	Indirect
Invasiveness	Non-invasive	Non-invasive	Invasive	Invasive	Non-invasive	Non-invasive	Invasive
Portability	Yes	No	Yes	Yes	No	Yes	No
Temporal resolution	~0.05 s	~0.05 s	~0.003 s	~0.003 s	~1 s	~1 s	1–2 min
Spatial resolution	~10 mm	~5 mm	~1 mm	~0.5 mm (LFP) ~0.1 mm (MUA) ~0.05 mm (SUA)	~1 mm	~5 mm	~4 mm
BCI applicability	Acceptable spatio-temporal resolution with high-density electrodes	Mobility constraint	Unfavorable for healthy BCI users	Unfavorable for healthy BCI users	Slow and mobility constraint	Slow, but mobile and a potential alternative to fMRI	Limited potentiality

*EEG, electroencephalography; MEG, magnetoencephalography; ECoG, electrocorticography; fMRI, functional magnetic resonance imaging; fNIRS, functional near infrared spectroscopy; PET, positron emission tomography*.

Recent technological advancements allow both the decoding of neural activities and the delivery of external signals into targeted brain areas to induce plasticity, i.e., remodeling of neurosynaptic organization (Lajoie et al., 2017). Plasticity is an inherent characteristic of the brain and peripheral nervous system underpinning BCI-based rehabilitation and other neuroscientific applications. While most of the BCI systems translate brain signals to computer commands, some systems utilize external stimulation modalities such as transcranial magnetic stimulation (Grau et al., 2014; Rao et al., 2014; Schaworonkow et al., 2019) and transcranial direct current stimulation (Baxter et al., 2017) to stimulate specific brain areas. The bidirectional framework of BCI comprises either one brain with feedback modality or two brains. Transcranial direct current stimulation directed by MI-related EEG signals alters the connectivity in sensorimotor networks of healthy individuals (Baxter et al., 2017). Another possible application of bidirectional BCI framework is direct brain-to-brain communication (Grau et al., 2014; Rao et al., 2014). Moreover, some BCI applications require auxiliary modalities, e.g., proprioceptive feedback and functional electrical stimulation driven by brain signals as feedback for augmenting or regaining peripheral motor actions (Darvishi et al., 2017; Bhattacharyya et al., 2019; Bockbrader et al., 2019; Murovec et al., 2020).

### 1.2. Factors Influencing BCI Performance

For medical applications of BCI, three criteria are essential: (1) a comfortable and convenient signal acquisition device, (2) system validation and dissemination, and (3) reliability and potentiality of BCI (Shih et al., 2012). For the restoration of mobility in patients with motor impairments, invasive intracortical recordings show better BCI performance (Hochberg, 2013) than non-invasive methods such as EEG (Milan and Carmena, 2010). The performance determines how efficiently a patient can perform an impaired motor task or communicate with an external device. Invasive modalities are also suitable for locked-in patients, because the benefits (significantly improved quality-of-life) outweigh the risks associated with implantation (Gilja et al., 2011). A pilot study found no adverse effects pertaining to surgery or tissue reaction at 1 year follow-up (Friehs et al., 2006). Invasive BCI should generally not be considered for neurologically intact people due to risks associated with surgery. However, invasive recordings may enable the utilization of localized inner cortex activities and a better interpretation of surface recordings from a non-invasive modality (Schalk, 2010; Lina et al., 2012).

Many factors influence BCI performance; taking the underlying cortical-subcortical networks into consideration is of crucial importance. For example, MI-induced signals are best recorded from premotor and motor areas, because premotor cortex, primary motor cortex and supplementary motor area along with basal ganglia and thalamus of the subcortical areas are the mostly activated areas during MI (Marchesotti et al., 2017). While EEG can capture premotor and motor area activation (Edelman et al., 2015; Saha et al., 2019a), intracortical electrodes can record signals from basal ganglia and thalamus (Sand et al., 2017).

Several issues can significantly impede BCI performance. Maintaining an acceptable signal-to-noise ratio in non-invasive long-term recordings is critical. Event-induced brain waves or oscillations are dynamic and affected by unstable resting state networks (RSNs) (Mantini et al., 2007). Time-variant psychophysiological (Gonçalves et al., 2006; Zhang et al., 2015; Acqualagna et al., 2016; Saha and Baumert, 2020), neuroanatomical (Kasahara et al., 2015) factors and users' fundamental traits (Ahn and Jun, 2015) cause unreliable estimates of RSNs, causing short and long-term signal variation within and across individuals (Saha and Baumert, 2020). Due to these intrinsic signal variations, BCI systems require subject-specific training, during which subjects attend a calibration session that is tedious and often frustrating. To eliminate subject-specific training, the concept of inter-subject associativity, demonstrated in previous works in case of natural vision (Hasson et al., 2004) and natural music listening (Abrams et al., 2013), could be exploited toward inter-subject operable BCI. Recent studies suggest that inter-subject operable sensorimotor rhythm-based BCI might become feasible for subjects who share common brain dynamics (Saha et al., 2017, 2018, 2019a; Saha and Baumert, 2020). Inter-subject BCI holds promise predominantly for healthy people and in applications such as gaming, drowsiness and lie detection, because rehabilitative BCI must consider the characteristics and severity of individual impairment (Park et al., 2016). Transfer learning can also reduce the effects of session-to-session and subject-to-subject variabilities, by using systems that were trained on data from different people exploiting commonalties and reducing training requirements (Jayaram et al., 2016; Saha et al., 2017, 2018, 2019a; He and Wu, 2019; Wu et al., 2020).

## 2. Challenges

### 2.1. Psychophysiological and Neurological Challenges

Emotional and mental processes, neurophysiology associated with cognition and neurological factors, i.e., functions, anatomy, play crucial roles in BCI performance and give rise to significant intra- and inter-individual variability (Saha and Baumert, 2020). Psychological factors such as attention, memory load, fatigue and competing cognitive processes (Gonçalves et al., 2006; Käthner et al., 2014; Calhoun and Adali, 2016) as well as users' basic characteristics such as lifestyle, gender, and age, (Kasahara et al., 2015) influence instantaneous brain dynamics. For example, individuals with lower empathy participate less emotionally in a P300-BCI paradigm and can produce higher amplitudes of P300 waves than subjects with greater empathetic involvement (Kleih and Kübler, 2013). Motivation is also related to P300-BCI performance (Nijboer et al., 2010).

Besides psychological traits, resting state physiological parameters, for example, frequency domain features of resting state heart rate variability are associated with BCI performance (Kaufmann et al., 2012). In addition, the baselines of RSNs are dynamic and modify any cortical signature instantaneously (Mantini et al., 2007). Age alters RSNs and associated cognitive responses (Wang et al., 2016b). Adapting to such time-variant RSNs is more demanding when the effects of RSNs mask event-related cortical responses (Jensen et al., 2011). Moreover, the inherent complexity and diversity in the formation of human brains (Sporns, 2013) that influence the functional neural networks (Honey et al., 2010), construct highly volatile neuronal connectivity over time and across subjects (Honey et al., 2009). An efficient BCI system must be robust to such inherent physiological fluctuations over time to enable more generalized systems (Saha and Baumert, 2020).

Experiments correlating BCI performance with neuroanatomical, neurophysiological and psychological parameters have provided fascinating results: gray matter volume in sensorimotor cortical areas is associated with BCI success (Kasahara et al., 2015). Sensorimotor rhythm-based BCI has implicated that physiological predictors such as spectral entropy and power spectral density, derived from resting state EEG recordings are correlated with BCI performance (Zhang et al., 2015; Acqualagna et al., 2016). Psychological predictors such as attention and motivation, are also associated with sensorimotor rhythm-based BCI performance (Hammer et al., 2012). Corticospinal excitability could be used as another reliable marker for BCI performance (Vasilyev et al., 2017). Taking head anatomy into consideration augments BCI performance (Wronkiewicz et al., 2015; Saha et al., 2019a).

Around 15–30% of individuals are inherently not able to produce brain signals robust enough to operate a BCI (Blankertz et al., 2009; Halder et al., 2019; Cecotti, 2020). Considering neurophysiological phenomena may reduce BCI illiteracy. An adaptive machine learning approach incorporating neurophysiological and psychological traits has been proposed to reduce BCI illiteracy (Vidaurre and Blankertz, 2010). The causes of BCI illiteracy do not exclusively rely on users' ability to produce signals. Sometimes technological limitations may hinder essential features extraction for a successful BCI operation for an individual. For example, measurements of scalp EEG/MEG may not show good task-specific signals due to the folding of the cortex or scalp-to-cortex distance for that individual (Andersen et al., 2020).

Other case-specific investigations on neuro-psycho-physiological parameters contributing to BCI performance are essential. For the rehabilitation of stroke survivors, affected neural circuits, i.e., lesions are to be identified carefully, because brain responses fluctuate according to the spatial location of the stroke lesion (Park et al., 2016). Although current neuroimaging methods are effective in capturing stroke lesion sites, a case-specific BCI design that incorporates residual brain function is required for rehabilitative interventions. Highly individualized design impedes wide dissemination of BCI-driven rehabilitation of neurological conditions.

### 2.2. Technological Challenges

Event related potential (ERP) (McCane et al., 2015), steady-state visual evoked potential (SSVEP) (Chen et al., 2015; Abu-Alqumsan and Peer, 2016), auditory evoked potential (AEP) (Schreuder et al., 2010), steady-state somatosensory evoked potential (SSSEP) (Muller-Putz et al., 2006; Oxley et al., 2017), and motor imagery (MI) (Marchesotti et al., 2016; Saha et al., 2019a; Saha and Baumert, 2020), have been proposed to detect cognitive signatures although none of the approaches performs well for all BCI applications. For example, ERPs and SSVEPs are target-specific and elicited by external stimuli; however, if ERPs depend on visual stimuli, they cannot be used for communication by locked-in patients with impaired visual processing. In that case, auditory-based ERP (e.g., AEP) could be used if auditory processing remains intact. The SSVEP method provides the highest information transfer rate of a non-invasive EEG-based BCI (Chen et al., 2015; Abu-Alqumsan and Peer, 2016). Limitations of the SSVEP technique include visual fatigue caused by looking at a flickering display for a long time. When using this technique, the control signal could be arbitrary and counter-intuitive, although it might depend mostly on the experimental context. For example, when using a BCI speller based on SSVEP, an individual looks at the letter “A”, which flickers at 10 Hz. It is generally not given importance to any inherent relationship between “A” and 10 Hz, instead the control signal is arbitrary mapped to interface with a computer. An advantage of an MI-based BCI is the use of explicit mapping of task-related brain signals to operate (Saha and Baumert, 2020). However, MI seems too slow for action control, thus they are not suitable for controlling virtual reality environments or videogames (Lécuyer et al., 2008). Recently proposed hybrid BCIs which utilize more than one signature, i.e., SSVEP/ERP (Combaz and Van Hulle, 2015; Yin et al., 2015) and SSVEP/MI (Pfurtscheller et al., 2010; Horki et al., 2011), seem to offer more robust features. Considering asynchronous BCI where the user decides to activate a command when necessary, the performance is still unsatisfactory (Han et al., 2020).

The intrinsic neurophysiological instability of brain dynamics poses critical challenges for making BCI systems efficient. The major components of a BCI system are signal acquisition, signal processing and effector device (Schwartz et al., 2006). Various neuroimaging techniques have been used to explore cortical activities through either electrical or hemodynamic signatures (Min et al., 2010), but none of the methods shows any advantage for a lucrative BCI design meeting the four important criteria: cost efficiency, portability, easy maintenance, and little or no involvement of surgery. EEG-based BCI are relatively more compliant with the abovementioned criteria as compared to other signal acquisition modalities. Tables 2, 3 list a diverse range of BCI applications exploiting EEG. Both invasive and non-invasive signal acquisitions have recently shown that reliable long-term (i.e., for at least several months) use of BCI systems is quite feasible (Saeedi et al., 2016; Sauter-Starce et al., 2019; Shahriari et al., 2019; Oxley et al., 2020).

**Table 2:** A summary of sensorimotor rhythms and electroencephalography-based brain computer interface (BCI) studies.

**References**	**Modality**	**Wave/Task**	**Data analysis**	**Context**	**Application**
Romero-Laiseca et al., 2020	EEG	MI	Riemannian geometry, LDA	Motor learning & plasticity induction	Lower limb rehabilitation
Song and Kim, 2019	EEG	MI	Spectral averaging	Visuo-Tactile stimulation	MI enhancement
Saha et al., 2019a	EEG	MI	wMEM	Transfer learning	Inter-subject BCI
Toriyama et al., 2018	EEG	MI, ME	LDA	MI & ME resemblance	Corticospinal excitability
Yu et al., 2018	EEG	MI	CSP, LDA	Assistive technology	Wheelchair navigation
Darvishi et al., 2017	EEG	MI	LF	Motor training by proprioceptive feedback	Upper limb rehabilitation
Park et al., 2016	EEG	MI, ME	ICA, CAR	Variable lesion characteristics	Stroke rehabilitation
Kasahara et al., 2015	EEG	MI	LF, AR	Neuroanatomical predictor	Motor rehabilitation
Edelman et al., 2015	EEG	MI	WMNE	Decoding complex MI	Higher degrees of freedom
Leamy et al., 2014	EEG	ME, MI	Filter bank CSP	Plasticity induction	Stroke rehabilitation
Ramos-Murguialday et al., 2013	EEG	ME, MI	AR	Stroke rehabilitation, assitive technology	Hand orthosis control
LaFleur et al., 2013	EEG	MI	AR	Assistive technology, robotics	Quadcopter control

*EEG, electroencephalography; ME, motor execution; MI, motor imagery; wMEM, wavelet-based maximum entropy on the mean; LF, Laplacian filter; AR, autoregressive model; CSP, common spatial pattern; ICA, independent component analysis; CAR, common average reference; WMNE, weighted minimum norm estimate; LDA, linear discriminant analysis*.

**Table 3:** A summary of non-sensorimotor rhythms and electroencephalography-based brain computer interface (BCI) studies.

**References**	**Modality**	**Wave**\**Task**	**Data analysis**	**Context**	**Application**
Jin et al., 2020	EEG	P300	LDA	Vibrotactile stimuli	Spinal cord injury rehabilitation
Cecotti, 2020	EEG	SSVEP	CCA	Generalized framework	BCI literacy
Halder et al., 2019	EEG	P300	LDA	Auditory stimuli	BCI literacy
Wei et al., 2018	EEG	Continuous (alert vs. drowsy)	PCA, LDA, SVM	Driving scenario in virtual reality	Drowsiness detection
Guy et al., 2018	EEG	P300	LDA	Amyotrophic lateral sclerosis	Visual P300 speller
Alcaide-Aguirre et al., 2017	EEG	P300	LDA	Cerebral palsy	Cognitive assessment
Waytowich et al., 2016	EEG	P300	Information geometry	Transfer learning	Inter-subject BCI
Norton et al., 2015	EEG	SSVEP, P300	Averaging	Soft, curved electrode systems	Text speller
Botrel et al., 2015	EEG	P300	LDA	Amyotrophic lateral sclerosis	Brain painting
Farwell et al., 2014	EEG	P300	Bootstrapping	Brain fingerprinting	Lie detection
Petrov et al., 2014	EEG	VEP	Naive Bayes	High-density EEG systems	Epilepsy or other BCI applications
Chi et al., 2011	EEG	SSVEP	CCA	Dry and non-contact sensors	Typical BCI application
Iturrate et al., 2009	EEG	P300	LDA	Assistive Technology	Wheelchair control

*EEG, electroencephalography; P300, an event-related potential; SSVEP, steady-state visual evoked potential; VEP, visual evoked potential; LDA, linear discriminant analysis; CCA, canonical correlation analysis; PCA, principal component analysis; SVM, support vector machine*.

EEG provides relatively poor spatial resolution due to non-invasive scalp recordings compared to fMRI, but finer temporal resolution (Lystad and Pollard, 2009; Min et al., 2010; He et al., 2011; Nicolas-Alonso and Gomez-Gil, 2012). Employing high density EEG mapping increases spatial resolution but results in high computational cost and efforts to maintain a reasonable signal-to-noise ratio across all channels (Chen et al., 2015). Since EEG captures only the electrical field associated cognitive processes, concomitant assessment of blood-oxygen level-dependent (BOLD) activity may improve BCI performance. BOLD activity is typically captured with fMRI (Sitaram et al., 2007), which is not feasible for most BCI applications, due to unmanageable size and cost of the device. fNIRS provides a safe, non-invasive, relatively inexpensive and portable neuroimaging alternative for recording BOLD activity (Matthews et al., 2007). Integrating fNIRS with EEG can significantly enhance classification performances regardless of low information transfer rate caused by inherent delays in hemodynamics (Fazli et al., 2012). A recent study has suggested that fNIRS is unable to adequately offer acceptable performances on its own, but can be combined with EEG to boost the performances (Ge et al., 2017). However, continuous technological advances could promote fNIRS as an exclusive tool for neuroscience research, including the development of BCI (Scholkmann et al., 2014; Naseer and Hong, 2015).

Probing sources in cortico-subcortical networks is another important limitation of scalp-based sensors such as EEG. Reconstructing task-induced networks while resolving the so-called inverse problem imposes a significant challenge. A two-equivalent-dipole model was applied on EEG data to discern the anatomical nature of the MI induced sources and to aid the classification performances (Kamousi et al., 2005). Saha et al. proposed a wavelet-based source localization approach to investigate MI-related sources and their impact on BCI performance (Saha et al., 2019a). The neuronal potentials attenuate through several tissue layers of complex geometry and diverse electrical properties; however, the magnetic permeability in the cerebrospinal fluid, skull, and skin, is consistent (da Silva, 2013). Thus, MEG can capture signal with less distortion than EEG. Although MEG provides better spatiotemporal resolution as compared to EEG, the magnetic field created by the brain is very small, requiring costly, stationary recording equipment (Mellinger et al., 2007; Corsi et al., 2019).

The BCI classifier design has to address two issues (Bashashati et al., 2007; Lotte et al., 2007, 2018). First, the dimensionality of the features set used for estimating the model parameters should be chosen for optimal performance based on the nature of the classifier. Second, the trade-off between bias and variance has to be considered and may involve regularizing the parameter estimation.

Covariate shift occurs when the features extracted from the training differ from those of test data impacting the classification performance (Krusienski et al., 2011). Covariate shift is an important issue requiring the application of adaptive methods for compensating feature space transitions (Jayaram et al., 2016; Saha and Baumert, 2020). The unsupervised subspace learning method enables session-to-session and subject-to-subject information transfers, augmenting BCI performance (Samek et al., 2013; Jayaram et al., 2016; Saha et al., 2018). The common spatial pattern, a supervised method, has been extensively used in EEG-based online and offline BCI settings (Ramoser et al., 2000; Wu et al., 2017a). A common problem with such a data-driven technique is over-fitting of the model parameters based on training sets, causing unreliable prediction on the test data (Sannelli et al., 2016). Recent studies integrated diverse methods into potential transfer learning frameworks for BCI including spatial filters (e.g., common spatial pattern), Riemannian geometry, Euclidean alignment and subspace adaptation and deep learning-based techniques (Barachant et al., 2011; Congedo et al., 2013, 2017; Marathe et al., 2015; Wu et al., 2016, 2017b; Wu et al., 2020; He and Wu, 2019, 2020; Kwon et al., 2019; Zhang and Wu, 2020).

## 3. Neuroplasticity, Sensors, Signal Processing, Modeling, and Applications

Exploiting neuroplasticity, designing hi-fidelity and customized neural sensors, applying advanced signal processing, and machine learning techniques are the key aspects of an effective BCI design. Tables 2–5 highlight diverse characteristics of BCI components and applications including signal acquisition modality, experimental paradigm, data analysis and pattern recognition, application field, and significance. Notably, there are no specific selection criteria for studies in Tables 2–5 due to the broad spectrum of topics covered in this review; however, they are summarized such that critical advances over the last several years can be appreciated.

### 3.1. Neuroplasticity and Cognitive Rehabilitation

The time-variant behavior of synapses within complex neural networks underpins the plastic characteristics of the brain and was first illustrated by Donald O. Hebb in 1949 (Brown and Milner, 2003). Neuroplasticity not only helps to assist cognitive and perceptual learning but also is the main ingredient for neurorehabilitation. How plastic a particular brain area is, may define the effectiveness of a neurofeedback strategy to induce specific activity patterns. Studies have shown visual cortices are plastic enough to produce robust neural signals for post-neurofeedback perceptual learning (Shibata et al., 2011; Amano et al., 2016). Another study has demonstrated if right/left hemispheric differences in neurofeedback-induced alpha activities are associated with visual information processing and motor behaviors, and, thus, control spatial attention (Jones and Sliva, 2020). fMRI-based neurofeedback training sessions induce the plasticity of attention-related behavior. Implications suggest that neurofeedback can offer rehabilitation of attentional deficit (Megan et al., 2015). A recent study has used neurofeedback to generate robust somatosensory oscillations associated with human perception (Brickwedde et al., 2019).

Closed-loop BCI with neurofeedback is assumed to contribute to the reorganization of cortical-subcortical neural networks and assist subjects in self-regulating specific brain rhythms; notwithstanding, the underlying mechanisms that alter neural substrates are still not fully-understood (Sitaram et al., 2017). For example, BCI-based covert visuomotor training modulates associated neural substrates, where the effects of modulated neural substrates are observed while performing that particular movement-related task (Vyas et al., 2018). Substantial changes in overt movement-related task following BCI-driven training induced learning suggest a critical role of BCI in enhanced motor learning for proficiently controlling neuroprosthetics (Orsborn et al., 2014), i.e., devices that can enhance or repair the output of the nervous system. For example, intracortical electrodes may be used to stimulate specific brain regions to regain motor control (Oxley et al., 2016; Sand et al., 2017). BCI may augment training-induced plasticity during therapeutic motor rehabilitation and, thus, re-excite corresponding neural substrates to regain control by means of neuroprosthetics or upper limb functions (Dobkin, 2007). Other examples include BCI-driven exoskeletons to enhance human working capacity (Benabid et al., 2019).

The extent of BCI-induced plasticity entails several factors, including (1) the selection of the signal acquisition modality, which plays an important role in diagnosing neural states, (2) the design of feedback modality that has explicit association with the neural signal classification performance, (3) the consideration of application-specific feedback delays, and (4) the utilization of a suitable feedback modality (Grosse-Wentrup et al., 2011). Neural ensemble recordings using signal acquisition modalities such as EEG, MEG, fNIRS, and fMRI have become dominant over single unit recordings. Behavioral activities are likely to be distributed across three-dimensional cortical-subcortical networks and that cannot be captured within single unit recordings (Nicolelis and Lebedev, 2009).

Rehabilitative BCI can be designed either by attaching neural prostheses to the impaired body parts or by re-stimulating the damaged synaptic networks; in any of the cases, the idea is to exploit and promote neuroplasticity (Wang et al., 2010; Ramos-Murguialday et al., 2013; Park et al., 2016; Darvishi et al., 2017; Toriyama et al., 2018; Song and Kim, 2019; Romero-Laiseca et al., 2020). In stroke patients with paretic muscles without residual finger movement, increased electromyographic activity post rehabilitation by BCI-driven orthoses exhibits increased neuromuscular coherence that is essential for restoring movement control (Pfurtscheller et al., 2000; Ramos-Murguialday et al., 2013). Explicit application of functional electrical stimulation regulated by EEG-based movement-related signatures further suggests a role of BCI in rehabilitation (Zhao et al., 2016). Increased electromyographic activity in paretic muscles is indicative of plasticity induced by electrical stimulation (De Marchis et al., 2016). For BCI-based rehabilitation in a real-life environment, differentiating between task-induced activities and resting state activities is a key factor for controlling the prosthesis or stimulation modality (Pahwa et al., 2015).

Externally stimulating the affected brain areas by electric or magnetic fields holds promise for stroke rehabilitation. A recent study demonstrated the induction of neuroplasticity in white matter and cortical functions in chronic stroke patients by motor imagery-based BCI and transcranial direct current stimulation applied to targeted brain areas (Hong et al., 2017). Magnetic stimulation of brain areas driven by BCI increases cortical activation in stroke patients (Johnson et al., 2018). The level of neuroplasticity achieved post-rehabilitation varies across subjects and, thus, an individual-specific training session is necessary (Leamy et al., 2014). The use of BCI-based motor rehabilitation for locked-in patients is limited because they are unable to fully interact with the system (Birbaumer and Cohen, 2007). Other examples of BCI-driven rehabilitations include optimizing the parameters for deep brain stimulation applied into the subthalamic nucleus in patients with Parkinson's disease (Sand et al., 2017) and treating major depressive disorder by BCI-driven transcranial magnetic stimulation (Ray et al., 2015).

Either by providing direct control of assistive technologies or by direct neurostimulation, BCI can help patients who may suffer from amyotrophic lateral sclerosis, cerebral palsy, brainstem stroke, spinal cord injuries, muscular dystrophies, or chronic peripheral neuropathies (Kauhanen et al., 2006; Iturrate et al., 2009; Mak and Wolpaw, 2009; Allison et al., 2010; Ramos-Murguialday et al., 2013; Leamy et al., 2014; Botrel et al., 2015; Combaz and Van Hulle, 2015; Edelman et al., 2015; Park et al., 2016; Zhao et al., 2016; Alcaide-Aguirre et al., 2017; Ge et al., 2017; Chiarelli et al., 2018; Guy et al., 2018; Yu et al., 2018; Rezazadeh Sereshkeh et al., 2019; Jin et al., 2020; Zuo et al., 2020). Providing auxiliary degrees of freedom improves the quality of life of people with disabilities significantly. Brain signals can be translated to drive wheelchairs (Galán et al., 2008; Iturrate et al., 2009; Perdikis et al., 2017; Tonin and Millán, 2020). Integration of BCI with a vision-guided autonomous system was shown to effectively perform the grasping task using a prosthetic arm in a tetraplegic patient (Downey et al., 2016). An implanted microelectrode array has been proposed to operate a three-dimensional neuroprosthetic device (Taylor et al., 2002).

### 3.2. Signal Acquisition, Signal Processing, and Modeling

A significant number of studies are now involved in combining multimodal signal acquisition modalities to augment current BCI systems. For example, simultaneous EEG and fMRI yield complementary features by exploiting good temporal resolution of EEG and good spatial resolution of fMRI (Debener et al., 2006). Enhanced multiclass sensorimotor tasks classification performance using hybrid EEG and fNIRS signals implicates the importance of features extracted from both hemodynamic and electrical activities (Buccino et al., 2016). MEG is another potential tool to combine with EEG, as it captures radially/tangentially dipole sources in cortical-subcortical networks and adds complementary information to EEG signals (Kauhanen et al., 2006). Skepticism might still present about the detection of brain activities originated from subcortical areas; however, an increasing number of studies argue that EEG and MEG could capture subcortical activities (Andersen et al., 2019; Min et al., 2020; Piastra et al., 2020). A recent trend is to combine different signal acquisition modalities together to improve BCI efficiency. Table 4 highlights multimodal and hybrid BCI applications.

**Table 4:** A summary of multimodal and hybrid brain computer interface (BCI) studies.

**References**	**Modality**	**Wave**\**Task**	**Data analysis**	**Context**	**Application**
Zuo et al., 2020	EEG	MI+P300	CSP, LDA	Hybrid BCI	Post-stroke rehabilitation
Rezazadeh Sereshkeh et al., 2019	EEG+fNIRS	Imagined speech	WT, LDA	Multimodal BCI	Robotic control
Corsi et al., 2019	EEG+MEG	MI	LDA	Multimodal BCI	Motor rehabilitation
Chiarelli et al., 2018	EEG+fNIRS	MI	Deep learning	Multimodal BCI	Motor rehabilitation
Ge et al., 2017	EEG+fNIRS	MI	CSP, SVM	Multimodal BCI	Motor rehabilitation
Zhao et al., 2016	EEG+FES	SSVEP	CSP	Motor plasticity	Paretic limb rehabilitation
Combaz and Van Hulle, 2015	EEG	P300+SSVEP	SVM	Hybrid BCI	Assistive control
Rao et al., 2014	EEG+TMS	Visuomotor	LF	Hyperinteraction	Brain-to-brain interface
Grau et al., 2014	EEG+TMS	MI	Spatial filter, re-referencing	Hyperinteraction	Brain-to-brain interface
Choi and Jo, 2013	EEG	P300+SSVEP+MI	CSP, CCA	Hybrid BCI-based assistive technology	Humanoid robot control
	EEG	SSVEP+MI	LDA	Hybrid BCI	Motor rehabilitation, assistive technology
Kauhanen et al., 2006	EEG+MEG	ME	Particle filters	Multimodal BCI	Spinal cord injury

*EEG, electroencephalography; fNIRS, functional near infrared spectroscopy; MEG, magnetoencephelography; FES, functional electrical stimulation; TMS, transcranial magnetic stimulation; P300, an event-related potential; SSVEP, steady-state visual evoked potential; MI, motor imagery; ME, motor execution; WT, wavelet transform; CSP, common spatial pattern; CCA, canonical correlation analysis; SVM, support vector machine; LDA, linear discriminant analysis; LF, Laplacian filter*.

The combination of signal processing and machine learning approaches plays critical role in translating any brain signal to a command for a computer or other external devices. Tables 2–5 highlight different signal processing and machine learning techniques. Representing signals in the time-frequency-space is necessary to obtain physiological correlates of BCI outcomes (McFarland et al., 2006; Bashashati et al., 2007). Fourier transform (FT) and autoregressive models are examples of time domain representations of brain signals while short time FT and wavelet transform are examples of time-frequency representations (McFarland et al., 2006; Bashashati et al., 2007). In case of spatial filtering, the most popular filtering approaches are common spatial pattern, independent component analysis and the Laplacian filter. A diverse range of inverse models allow to discern the actual sources projected on three-dimensional cortical-subcortical networks (Wronkiewicz et al., 2015; Saha et al., 2019a). Extracted features can be translated using various linear and non-linear classification algorithms. Examples of linear and non-linear classifier models are linear discriminant analysis and non-linear kernel-based support vector machines (Lotte et al., 2007, 2018).

**Table 5:** A summary of brain computer interface (BCI) studies involving invasive procedures.

**References**	**Modality**	**Wave**\**Task**	**Data analysis**	**Context**	**Application**
Oxley et al., 2020 Oxley et al., 2017 Oxley et al., 2016	Stentrode	SSSEP	Spectral analysis	Catheter angiography- guided implantation	Minimally invasive BCI: human clinical trial in progress
Sauter-Starce et al., 2019 Mestais et al., 2014	ECoG	SSEP	Spectral analysis	WIMAGINE (Wireless Implantable Multi-channel Acquisition system for Generic Interface with Neurons)	Intracranial BCI: validation on *in vivo* sheep model
Sand et al., 2017	EEG	DBS response	Averaging	Parkinson's disease	Motor rehabilitation
Kaiju et al., 2017	ECoG	SEP	Wavelet transform	Finger stimulation	Motor learning/ rehabilitation
Vansteensel et al., 2016	iMEA	Visuomotor	Autoregression filter	Late-stage amyotrophic lateral sclerosis	Motor rehabilitation
Downey et al., 2016	iMEA	ME	Firing rate estimator	Vision-guided assistive technology	Robotic prosthetics control
Pahwa et al., 2015	ECoG	Sleep-Wake	Welch's periodogram, logistic regression	Assistive technology	Neuroprosthetic control
Yin et al., 2014	iMEA	ME, Visuomotor, Sleep-Wake	PCA	Full-spectrum electrophysiological recording	BCI, diagnostics, therapeutic treatments
Keefer et al., 2008	iMEA	Video watching	Spectrogram	Carbon nanotube- coated electrodes	Neural decoding and stimulation
Friehs et al., 2006	iMEA	MI	Not specified	Assistive technology/ clinical use of BCI	Cursor control/ epilepsy monitoring

*ECoG, electrocorticography; iMEA, intracortical microelectrode array; Stentrode, stent-electrode recording array; EEG, electroencephelography; ME, motor execution; SEP, somatosensory evoked potential; MI, motor imagery; SSSEP, steady-state somatosensory evoked potential; DBS, deep brain stimulation; PCA, principal component analysis*.

Since the first publication in 2000, common spatial pattern is still one of the most popular methods to represent multichannel EEG signals by corresponding spatial contents (Ramoser et al., 2000). As a data-driven method, it requires a significant number of training samples to model the filtering parameters. In case of small training trials, regularizing the covariance estimation works better than the traditional algorithm (Lotte and Guan, 2010). Other modifications in spatial filtering include projecting EEG by using sparse representation and filter bank spectral division of raw signals (Arvaneh et al., 2014). Generally, spatial filtering is applicable in subject-specific BCI development although recent studies have proposed estimating the filter coefficients from a subject and applied that filter to another subject, which contributed no training sample (Saha et al., 2017, 2018, 2019a). Other popular data-driven methods include linear discriminant analysis, support vector machine and principal component analysis (Lotte et al., 2007, 2018). With the exceptional advancements in computational facilities in the last decade, deep learning-based BCI paradigms by allowing the evaluation of large datasets could soon become a trend in the community (Chiarelli et al., 2018; Kwon et al., 2019; Nagel and Spüler, 2019).

On the other hand, independent component analysis is a blind source separation method requiring no training. The estimation of independent components is based on statistical properties of the signals (Bell and Sejnowski, 1995). However, modeling the actual cortical sources as dipoles in the complex brain anatomy from the scalp EEG recordings seeks to solve the so-called inverse problem (Qin et al., 2004; Kamousi et al., 2005; Wronkiewicz et al., 2015; Saha et al., 2019a). More recent source localization methods such as wavelet-based maximum entropy on the mean represent EEG/MEG signals as relevant time-frequency contents and finally transform them into spatial representations (Lina et al., 2012; Saha et al., 2019a). Notably, different inverse methods and toolboxes demonstrate considerable variability in localized sources (Mahjoory et al., 2017). Even it is not very straightforward to know the exact sources, which are to be modeled using EEG/MEG. For example, the ground truth defined by implanted electrodes might not be 100% reliable because of sparse (spatial) sampling. In the case of fMRI, the measurement of neural activity is indirect. Notwithstanding, inverse methods have shown promise for designing various BCI models (Qin et al., 2004; Kamousi et al., 2005; Wronkiewicz et al., 2015; Saha et al., 2019a).

### 3.3. Neurosensors: The-State-of-the-Art

Deeper regions of the brain, e.g., subcortical and cerebellar regions, contribute to various neuronal activities (Müller et al., 2002; Wardman et al., 2014). Interpreting the genesis of cortical sources from cellular to scalp levels and RSNs spanned throughout the three-dimensional brain space can guide BCI development (Donoghue, 2008). Sensors with customized design are developed to advance brain signal acquisition modalities. Neurosensors can be constructed in different forms like electrical, optical, chemical and biological (Deisseroth and Schnitzer, 2013). Dry EEG electrodes are convenient, but assumed to provide lower signal-to-noise ratio compared to conventional wet electrodes. Wet electrodes may cause inconvenience to users as they use conductive gel and require proper skin preparation for minimizing the skin-electrode impedance (Liao et al., 2012). However, a study on dry electrodes-based BCI suggested that dry electrode could be used to collect good quality signals by designing the circuits carefully (Chi et al., 2011). Further studies support dry electrodes with wireless systems that could offer comparable signal quality as of wet electrodes, but with more convenience (Di Flumeri et al., 2019; Marini et al., 2019; Hinrichs et al., 2020). While utilizing the advantages of both dry and wet electrodes, quasi-dry electrodes exploiting the mechanical properties of polymer can capture signals as comparable to commercial Ag/AgCl electrodes (Mota et al., 2013). To increase the spatial resolution of EEG, Petrov et al. have proposed an ultra-dense sensor array of 700–800 electrodes (Petrov et al., 2014). The signal-to-noise ratio was twice as high as for high-density EEG that has up to 256 gold-coated electrodes. An auricle electrode with stretchable connector was proposed that not only can increase portability but also can offer a comfortable alternative for long term recordings (Norton et al., 2015). The electrode is flexible with the alterations of electrical and mechanical properties of skin.

Invasive sensors must be biocompatible. A novel organic electrochemical transistor-based sensor enables to collect neural signals directly from the brain surface (Khodagholy et al., 2013). This sensor is biocompatible and mechanically flexible, and the transistor-based design amplifies captured signals locally, thus providing much better signal-to-noise ratio than conventional ECoG. To enhance the signal quality, carbon nanotube coating can decrease the electrode impedance and, thus, increase the charge transfer (Keefer et al., 2008). Another invasive biocompatible sensor, designed for recording previously inaccessible spectra of large neuron populations, includes data transmission for use in natural environments (Yin et al., 2014). With the outstanding progress of nanotechnology, nanowire Field Effect Transistor and other p/n junction devices have potential for neuro-sensing modalities for intracellular recordings, even in the deep brain regions (Kruskal et al., 2015). Oxley et al. have proposed stent-electrode array (stentrode) that involves minimal invasiveness (Oxley et al., 2016, 2017). Using computer-guided catheter angiography, the stentrode can be placed within arteries or veins located inside the brain anatomy. Capturing high-fidelity cortical signals, this technology will significantly reduce the risk factors of craniotomy. A follow-up study has recently demonstrated successful implantation of the strentrode in humans for long-term neural signal recording (Oxley et al., 2020). The information transfer rate for strentrode-based BCI was comparable to the landmark study by Vansteensel et al. with implanted electrodes (Vansteensel et al., 2016). Another implanted ECoG recorder called as WIMAGINE (Wireless Implantable Multi-channel Acquisition system for Generic Interface with Neurons) allows wireless neural data access (Mestais et al., 2014). The WIMAGINE has recently been tested for long-term reliability of data acquisition and any risk associated with craniotomy (Kruskal et al., 2015; Sauter-Starce et al., 2019).

Besides large-scale recording modalities like EEG and MEG, very small-scale recordings of neuronal activities are crucial for understanding brain circuits' functions and intra- and inter-neuron interactions. Representation of any cognitive task as functions of both small-scale and large-scale neuronal interactions is crucial for the advancement of neuroengineering and BCI. In this regard, a high-density neurosensor array made from silicon probes combined with optogenetics enables single unit recordings (Buzsáki et al., 2015). Yang et al. proposed a novel multi-plane two-photon microscope that can be used to capture multi-layer neuronal structure and mechanism with cellular resolution (Yang et al., 2016). Other potential imaging methods for investigating cell signaling include calcium imaging (Grienberger and Konnerth, 2012) and advanced microscope with chronically implanted lenses (Resendez et al., 2016). Designer receptor exclusively activated by designer drugs, provides a chemogenetic tool to understand cell-signaling including electrical activities in molecularly clustered cell groups (Sternson and Roth, 2014; Roth, 2016). A new ultrasonic-based wireless system, called neural dust, enables the recording of electromyogram and electroneurogram on the millimeter scale (Seo et al., 2016).

### 3.4. Affective Computing, Gaming, Robotics, and Miscellaneous Applications

Future computers are assumed to have emotional and perceptual capabilities, which could extend the use not only to assisting humans but also to making decisions (Picard, 2000). Computers might able to recognize and interpret underlying affective states based on physiological and behavioral variables. Recent studies demonstrated BCI is a potential tool to investigate affective states, expanding the applications into psychology (Piho and Tjahjadi, 2018; Song et al., 2018; Huang et al., 2019). Huang et al. have proposed an EEG-based BCI to detect positive and negative emotions induced by video stimulus (Huang et al., 2019).

Integration of arts into BCI is referred to as artistic BCI (Andujar et al., 2015). In the late 1960s, David Rosenboom began experimenting with ways to link brain functions with musical production, perception of musical forms and musical proprioception (Rosenboom, 2014). Other examples of artistic BCI include affective states detection, playing video games and controlling virtual/augmented reality environment. Studies have demonstrated that a user can fully operate video games by SSVEP-BCI (van Vliet et al., 2012; Filiz and Arslan, 2020). Other studies have proposed how multiple users can participate in a collaborative game, in which joint decision making is required to control the gaming environment (Nijholt and Poel, 2016; Sekhavat, 2020). Another study previously suggested the aggregation of information from two intelligence analysts' brain signals may lead to better decision making than one's brain signals (Stoica, 2012). The underlying cause could be explained by inter-individual differences in human cognitive and perceptual skills (Kleinschmidt et al., 2012). Collaboration between users might assist an individual's decision making by diversity inclusion. A modified setup could investigate how people interact in different social contexts, extending BCI applications in sociology (Amaral et al., 2017).

Virtual/augmented reality (V/AR) technologies together with BCI could offer immersive experiences and have many potential applications including arts and neurofeedback (Andujar et al., 2015; Tremmel et al., 2019; Putze et al., 2020). Brain painting allows a user to draw lines in a virtual canvas by brain signals, which gives an alternative communication channel for people with paretic motor functions (Botrel et al., 2015). McClinton et al. have developed a brain painting application using VR environment (McClinton et al., 2019). Another work has evinced VR-BCI to measure cognitive workload that can contribute to neuroergonomics (Tremmel et al., 2019). Studies have also used immersive VR as a better neurofeedback option as compared to the computer screen leading to increased BCI accuracy (Luu et al., 2016; Škola et al., 2019; Vourvopoulos et al., 2019; Juliano et al., 2020). Vourvopoulos et al. have integrated the principles of VR and BCI into a platform called REINVENT for motor rehabilitation (Vourvopoulos et al., 2019). Likewise, BCI with AR can be used to remotely control a robot for rehabilitating children with attention-deficit/hyperactivity disorder (Arpaia et al., 2020).

While BCI-driven robotic controllers can offer advanced assistive technology for people with mobility constraint, it may also augment human ergonomic performance for healthy subjects (Millan et al., 2004; Gandhi et al., 2014; Tidoni et al., 2016; Perdikis et al., 2017; Spataro et al., 2017; Yuan and Li, 2018; Deng et al., 2019; Tonin et al., 2019; Tonin and Millán, 2020). EEG-based BCI-driven controller of mobile robot or wheelchair has demonstrated the possibility of this technology in robotics industry (Millan et al., 2004; Tidoni et al., 2016; Perdikis et al., 2017; Yuan and Li, 2018; Deng et al., 2019; Tonin et al., 2019). BCI can also be used for controlling humanoid robots remotely using EEG (Spataro et al., 2017), suitable in hazardous environments, for example by sending a robot in a coal mine for executing a task that is potentially unsafe for a human. In space, BCI can be used to monitor astronauts' working capacity and to drive an exoskeleton (Menon et al., 2009; de Negueruela et al., 2011). In the absence of gravity, working becomes tedious and inconvenient. Furthermore, astronauts' working time is precious. BCI-driven systems could be practical for improving astronauts' functionality, efficiency and safety (Summerer et al., 2009; Farwell et al., 2014; Botrel et al., 2015; Ortiz et al., 2016; Wang et al., 2016a; Vourvopoulos et al., 2019; Singh et al., 2020).

**Figure 2:**
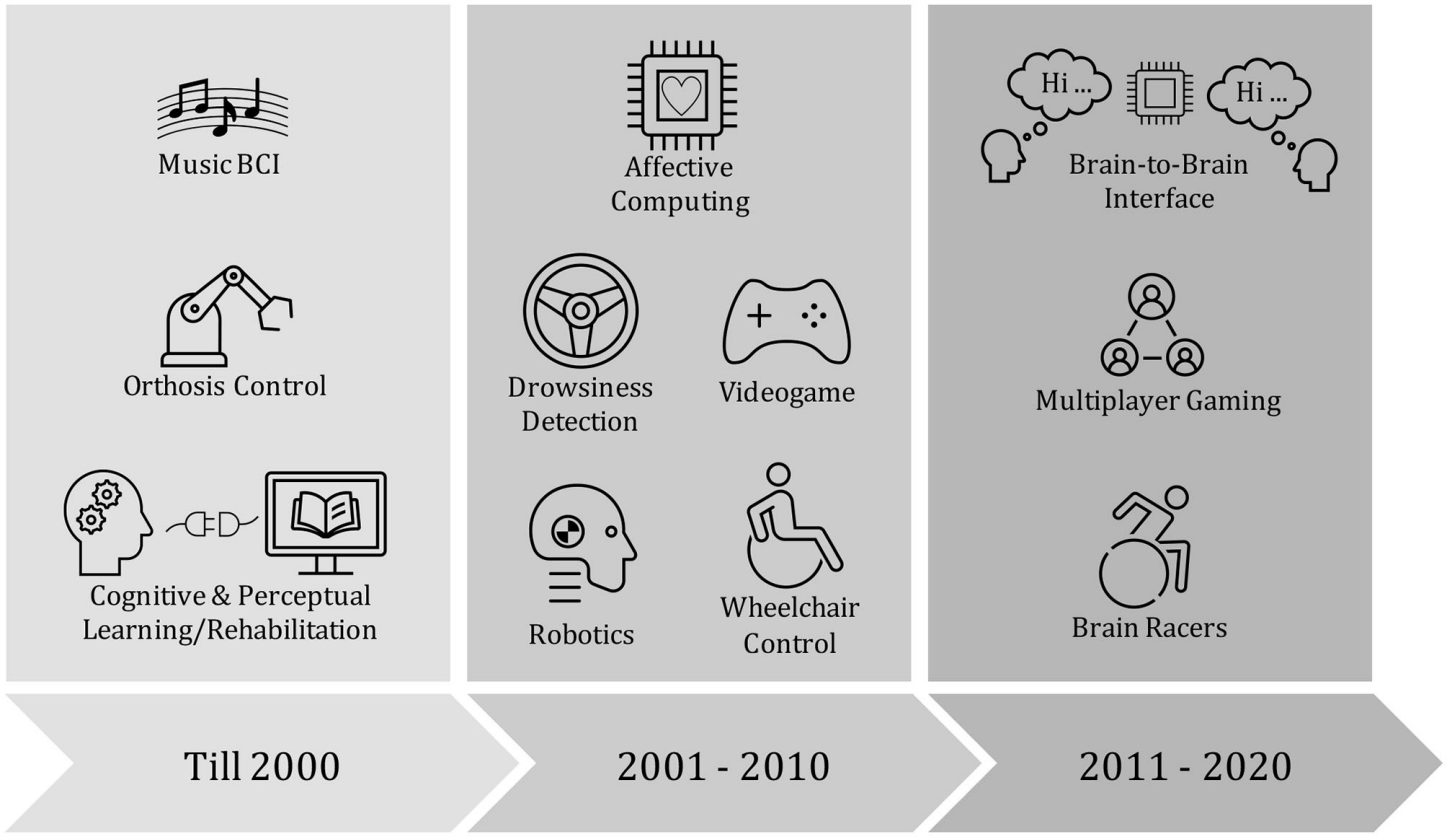
A schematic illustration of the evolution of the brain computer interface (BCI) applications: Cognitive & Perceptual Learning/Rehabilitation (McMillan et al., 1995); Orthosis Control (Pfurtscheller et al., 2000); Music BCI (Rosenboom, 2014); Robotics (Millan et al., 2004); Wheelchair Control (Iturrate et al., 2009); Drowsiness Detection (Lin et al., 2008); Affective Computing (Zander et al., 2009); Brain Racers (Perdikis et al., 2017); Multiplayer Gaming (Nijholt and Poel, 2016); Brain-to-Brain Interface (Rao et al., 2014).

Recently, brain-to-brain interface (BBI) experiments that involve decoding sender's cognitive intentions, translate them into commands for stimulating receiver's brain, have been explored (Pais-Vieira et al., 2013; Rao et al., 2014; Jiang et al., 2019). In 2013, researchers implemented a direct BBI system in which one rat was able to share sensorimotor information to another rat (Pais-Vieira et al., 2013). Intracortical microstimulation was used to stimulate the receiver's target brain areas. An early attempt to develop sensorimotor rhythm-based BBI between two human subjects used non-invasive EEG and transcranial magnetic stimulation has been proposed by Rao et al. (2014). Other total non-invasive BBI experiments have proposed sharing pseudo-random binary streams encoded words between human subjects (Grau et al., 2014) and playing collaborative games (Stocco et al., 2015). Figure 2 illustrates a timeline for current advances of BCI in diverse applications.

## 4. Ethical Concerns and Socioeconomic Contexts

Irrespective of the scientific breakthroughs in BCI field, there are key factors pertaining to safety, ethics, privacy protection and data confidentiality, community acceptance and socioeconomic aspects that should be considered with adequate precautions to maximize users' benefits and social impacts (Illes and Bird, 2006; Bostrom and Sandberg, 2009; Jebari, 2013; McCullagh et al., 2014). Obtaining an ethically sound informed consent from a BCI worn patient may be challenging for BCI researchers due to difficulty in communicating and the lack of alternatives. However, more awareness and attention to ethics policies are recommended to improve the chance for patients to get adequate information.

Physical and mental safety of BCI users is important. Invasive procedures such as deep brain stimulation and intracortical microelectrode array may cause postoperative psychological and neurological side effects (Jotterand and Giordano, 2011; Gilbert, 2015; Maslen et al., 2015). Additionally, bleeding and infections are infrequent but do occur and may require removal or further maintenance of the implanted electrodes. Guidelines are required to safely advance neurotechnologies (Goering and Yuste, 2016), because BCI devices can alter behavior and, thus, introduce potential threats to one's emotions, personality and memories; more generally one's mind. For human brain-to-brain interface applications (Rao et al., 2014; Stocco et al., 2015), one may define an upper bound for research depths keeping in mind the necessity of ethical utilization of this technology. Because both sender and receiver play complicated roles, more specifically, sender's intentional manipulative control over neural signals might alter the anticipated outcome. Altering human cognitive and possibly moral capacity raise a serious ethical question and it is not predictable if the cognitive changes reversible and efficacious (Nakazawa et al., 2016).

A user's expectations of achieving extended or auxiliary degree of freedom may not be fulfilled, and even the unfamiliar risk factors can diminish the accomplished advantage of using BCI (Clausen, 2011; Schicktanz et al., 2015). Creating broad awareness of BCI technology and its pros and cons would educate people, who fear unnecessary technological dependency (Hobson et al., 2017). However, successful clinical trials of sophisticated devices such as strentrode or WIMAGINE are essential to demonstrate potential advantages, especially for people suffering from any form of cognitive disability (Sauter-Starce et al., 2019; Oxley et al., 2020). In the case of healthy users, it should not be too difficult to create acceptance to a broader community when dry electrodes could offer the long-term operation of a BCI application with little maintenance effort (Di Flumeri et al., 2019; Marini et al., 2019; Hinrichs et al., 2020).

It is critical to introduce a suitable act for lawful utilization of BCI and preservation of privacy and confidentiality of stored data. Recent studies have demonstrated decoding of password or recognizing faces utilizing consumer-grade BCI successfully, prompting a potential concern of any illegitimate access to users' raw data and their further exploitation (Martin et al., 2016; Alomari et al., 2019). For example, affective states define users' moral judgment and emotional traits. Thus, it is critical to limit the applications of affective BCI while preserving sensitive information (Steinert and Friedrich, 2020). Necessary precursor initiatives should propose application-specific BCI frameworks, which can restrict unauthorized access to stored data or the system (Ienca and Haselager, 2016). For example, illicit access to a wireless BCI-driven limb and manipulative reprogramming of a computer-guided neuro-stimulation have demonstrated the importance of establishing resilient safeguards to BCI use (Denning et al., 2009). Agarwal et al. have proposed cryptographic protocols as integrated parts of BCI to preserve the privacy of a user by keeping confidential information obscure to others (Agarwal et al., 2019). Without evaluating socioeconomic, ethical and policy issues, the commercialization of BCI would hinder the progress in this field (Eaton and Illes, 2007).

By creating a common networking platform for BCI researchers worldwide, the immediate proposition of a comprehensive list of universal guidelines is key to sustainable advancements of the field (Vaadia and Birbaumer, 2009). Various alliance-based projects are running as common platforms for advancing the knowledge of neuroscience, for example, by strengthening efforts to fund neuroscience research projects (Grillner et al., 2016). The European Union along with its partner universities have initiated the Human Brain Project. In addition, the Brain Initiative has been announced by the White House. In our opinion, advanced understanding of basic neuroscientific phenomena will determine the structure, efficacy and applications of futuristic BCI.

## 5. Conclusion

Numerous groundbreaking advances in neurosensors and computational tools herald great promise for more sophisticated and user friendly BCI systems requiring no or little maintenance. In addition to hi-fidelity signal acquisition, significant progress in signal processing and machine learning tools, their complementary roles, and high computation power and increased mobility of computers have significantly contributed in the emergence of BCI technologies. The future of BCI technology will rely greatly on addressing the following key aspects:

- Elucidating the underlying psychophysiological and neurological factors that potentially influence BCI performance.
- Designing less invasive sensors with reliable signal acquisition and resolution, while considering portability, easy maintenance, and affordability.
- Modeling session-to-session and subject-to-subject information transfer for the proposition of more generalized BCI models with insignificant or no calibration requirement.
- Establishing broad consensus on ethical issues and beneficial socioeconomic application of this technology.

## Author Contributions

SS conceived the initial idea, wrote the first draft, and generated all figures and tables. KM, KA, RM, GN, and SD participated in the discussion and commented on the draft. AHK and MB provided further insight and helped SS to finalize the structure and materials. All authors read and approved the final paper.

## Conflict of Interest

The authors declare that the research was conducted in the absence of any commercial or financial relationships that could be construed as a potential conflict of interest.

## References

[B1] Abrams D. A. Ryali S. Chen T. Chordia P. Khouzam A. Levitin D. J.. (2013). Inter-subject synchronization of brain responses during natural music listening. Eur. J. Neurosci. 37, 1458–1469. 10.1111/ejn.12173 23578016 PMC4487043

[B2] Abu-Alqumsan M. Peer A. (2016). Advancing the detection of steady-state visual evoked potentials in brain-computer interfaces. J. Neural Eng. 13:036005. 10.1088/1741-2560/13/3/036005 27064728

[B3] Acqualagna L. Botrel L. Vidaurre C. Kübler A. Blankertz B. (2016). Large-scale assessment of a fully automatic co-adaptive motor imagery-based brain computer interface. PLoS ONE 11:e0148886. 10.1371/journal.pone.0148886 26891350 PMC4758702

[B4] Agarwal A. Dowsley R. McKinney N. D. Wu D. Lin C.-T. De Cock M.. (2019). Protecting privacy of users in brain-computer interface applications. IEEE Trans. Neural Syst. Rehabil. Eng. 27, 1546–1555. 10.1109/TNSRE.2019.2926965 31283483

[B5] Ahn M. Jun S. C. (2015). Performance variation in motor imagery brain-computer interface: a brief review. J. Neurosci. Methods 243, 103–110. 10.1016/j.jneumeth.2015.01.033 25668430

[B6] Alcaide-Aguirre R. Warschausky S. Brown D. Aref A. Huggins J. (2017). Asynchronous brain-computer interface for cognitive assessment in people with cerebral palsy. J. Neural Eng. 14:066001. 10.1088/1741-2552/aa7fc4 28981448

[B7] Allison B. Z. Brunner C. Kaiser V. Müller-Putz G. R. Neuper C. Pfurtscheller G. (2010). Toward a hybrid brain-computer interface based on imagined movement and visual attention. J. Neural Eng. 7:026007. 10.1088/1741-2560/7/2/026007 20332550

[B8] Alomari R. Martin M. V. MacDonald S. Maraj A. Liscano R. Bellman C. (2019). Inside out-a study of users' perceptions of password memorability and recall. J. Inform. Security Appl. 47, 223–234. 10.1016/j.jisa.2019.05.009

[B9] Amano K. Shibata K. Kawato M. Sasaki Y. Watanabe T. (2016). Learning to associate orientation with color in early visual areas by associative decoded fMRI neurofeedback. Curr. Biol. 26, 1861–1866. 10.1016/j.cub.2016.05.014 27374335 PMC4961545

[B10] Amaral C. P. Simões M. A. Mouga S. Andrade J. Castelo-Branco M. (2017). A novel brain computer interface for classification of social joint attention in autism and comparison of 3 experimental setups: a feasibility study. J. Neurosci. Methods 290, 105–115. 10.1016/j.jneumeth.2017.07.029 28760486

[B11] Andersen L. M. Jerbi K. Dalal S. S. (2019). Can electro-and magnetoencephalography detect signals from the human cerebellum? PeerJ 7:e27901. 10.7287/peerj.preprints.27901 PMC7306153 32278092

[B12] Andersen L. M. Jerbi K. Dalal S. S. (2020). Can EEG and MEG detect signals from the human cerebellum? Neuroimage 215:116817. 10.1016/j.neuroimage.2020.116817 32278092 PMC7306153

[B13] Andujar M. Crawford C. S. Nijholt A. Jackson F. Gilbert J. E. (2015). Artistic brain-computer interfaces: the expression and stimulation of the user's affective state. Brain Comput. Interfaces 2, 60–69. 10.1080/2326263X.2015.1104613

[B14] Aricó P. Borghini G. Di Flumeri G. Colosimo A. Bonelli S. Golfetti A.. (2016). Adaptive automation triggered by EEG-based mental workload index: a passive brain-computer interface application in realistic air traffic control environment. Front. Hum. Neurosci. 10:539. 10.3389/fnhum.2016.00539 27833542 PMC5080530

[B15] Arpaia P. Duraccio L. Moccaldi N. Rossi S. (2020). Wearable brain-computer interface instrumentation for robot-based rehabilitation by augmented reality. IEEE Trans. Instrument. Meas. 69, 6362–6371. 10.1109/TIM.2020.2970846

[B16] Arvaneh M. Guan C. Ang K. K. Quek C. (2014). Mutual information-based optimization of sparse spatio-spectral filters in brain-computer interface. Neural Comput. Appl. 25, 625–634. 10.1007/s00521-013-1523-7

[B17] Barachant A. Bonnet S. Congedo M. Jutten C. (2011). Multiclass brain-computer interface classification by riemannian geometry. IEEE Trans. Biomed. Eng. 59, 920–928. 10.1109/TBME.2011.2172210 22010143

[B18] Bashashati A. Fatourechi M. Ward R. K. Birch G. E. (2007). A survey of signal processing algorithms in brain-computer interfaces based on electrical brain signals. J. Neural Eng. 4:R32. 10.1088/1741-2560/4/2/R03 17409474

[B19] Baxter B. S. Edelman B. J. Sohrabpour A. He B. (2017). Anodal transcranial direct current stimulation increases bilateral directed brain connectivity during motor-imagery based brain-computer interface control. Front. Neurosci. 11:691. 10.3389/fnins.2017.00691 29270110 PMC5725434

[B20] Bell A. J. Sejnowski T. J. (1995). An information-maximization approach to blind separation and blind deconvolution. Neural Comput. 7, 1129–1159. 10.1162/neco.1995.7.6.1129 7584893

[B21] Benabid A. L. Costecalde T. Eliseyev A. Charvet G. Verney A. Karakas S.. (2019). An exoskeleton controlled by an epidural wireless brain-machine interface in a tetraplegic patient: a proof-of-concept demonstration. Lancet Neurol. 18, 1112–1122. 10.1016/S1474-4422(19)30321-7 31587955

[B22] Berger H. (1929). Über das elektroenkephalogramm des menschen. Arch. Psychiatr. Nervenkrankheiten 87, 527–570. 10.1007/BF01797193

[B23] Bhattacharyya S. Clerc M. Hayashibe M. (2019). Augmenting motor imagery learning for brain-computer interfacing using electrical stimulation as feedback. IEEE Trans. Med. Robot. Bionics 1, 247–255. 10.1109/TMRB.2019.2949854

[B24] Birbaumer N. Cohen L. G. (2007). Brain-computer interfaces: communication and restoration of movement in paralysis. J. Physiol. 579, 621–636. 10.1113/jphysiol.2006.125633 17234696 PMC2151357

[B25] Birbaumer N. Ghanayim N. Hinterberger T. Iversen I. Kotchoubey B. Kübler A.. (1999). A spelling device for the paralysed. Nature 398, 297–298. 10.1038/18581 10192330

[B26] Blankertz B. Sanelli C. Halder S. Hammer E. Kübler A. Müller K.-R.. (2009). Predicting BCI performance to study BCI illiteracy. BMC Neurosci. 10(Suppl. 1):P84. 10.1186/1471-2202-10-S1-P8424551050

[B27] Bockbrader M. Annetta N. Friedenberg D. Schwemmer M. Skomrock N. Colachis I. V.. (2019). Clinically significant gains in skillful grasp coordination by an individual with tetraplegia using an implanted brain-computer interface with forearm transcutaneous muscle stimulation. Arch. Phys. Med. Rehabil. 100, 1201–1217. 10.1016/j.apmr.2018.07.445 30902630

[B28] Bostrom N. Sandberg A. (2009). Cognitive enhancement: methods, ethics, regulatory challenges. Sci. Eng. Ethics 15, 311–341. 10.1007/s11948-009-9142-5 19543814

[B29] Botrel L. Holz E. Kübler A. (2015). Brain painting v2: evaluation of p300-based brain-computer interface for creative expression by an end-user following the user-centered design. Brain Comput. Interfaces 2, 135–149. 10.1080/2326263X.2015.1100038

[B30] Brickwedde M. Krüger M. C. Dinse H. R. (2019). Somatosensory alpha oscillations gate perceptual learning efficiency. Nat. Commun. 10, 1–9. 10.1038/s41467-018-08012-0 30651567 PMC6335466

[B31] Brown R. E. Milner P. M. (2003). The legacy of Donald O. Hebb: more than the Hebb synapse. Nat. Rev. Neurosci. 4, 1013–1019. 10.1038/nrn1257 14682362

[B32] Brunner C. Birbaumer N. Blankertz B. Guger C. Kübler A. Mattia D.. (2015). BNCI horizon 2020: towards a roadmap for the BCI community. Brain Comput. Interfaces 2, 1–10. 10.1080/2326263X.2015.1008956

[B33] Buccino A. P. Keles H. O. Omurtag A. (2016). Hybrid EEG-fNIRS asynchronous brain-computer interface for multiple motor tasks. PLoS ONE 11:e0146610. 10.1371/journal.pone.0146610 26730580 PMC4701662

[B34] Buzsáki G. Stark E. Berényi A. Khodagholy D. Kipke D. R. Yoon E.. (2015). Tools for probing local circuits: high-density silicon probes combined with optogenetics. Neuron 86, 92–105. 10.1016/j.neuron.2015.01.028 25856489 PMC4392339

[B35] Calhoun V. D. Adali T. (2016). Time-varying brain connectivity in fMRI data: whole-brain data-driven approaches for capturing and characterizing dynamic states. IEEE Signal Process. Mag. 33, 52–66. 10.1109/MSP.2015.2478915

[B36] Cecotti H. (2020). Adaptive time segment analysis for steady-state visual evoked potential based brain-computer interfaces. IEEE Trans. Neural Syst. Rehabil. Eng. 28, 552–560. 10.1109/TNSRE.2020.2968307 31985428

[B37] Chen X. Wang Y. Nakanishi M. Gao X. Jung T.-P. Gao S. (2015). High-speed spelling with a noninvasive brain-computer interface. Proc. Natl. Acad. Sci. U.S.A. 112, E6058–E6067. 10.1073/pnas.1508080112 26483479 PMC4640776

[B38] Chi Y. M. Wang Y.-T. Wang Y. Maier C. Jung T.-P. Cauwenberghs G. (2011). Dry and noncontact EEG sensors for mobile brain-computer interfaces. IEEE Trans. Neural Syst. Rehabil. Eng. 20, 228–235. 10.1109/TNSRE.2011.2174652 22180514

[B39] Chiarelli A. M. Croce P. Merla A. Zappasodi F. (2018). Deep learning for hybrid EEG-fNIRS brain-computer interface: application to motor imagery classification. J. Neural Eng. 15:036028. 10.1088/1741-2552/aaaf82 29446352

[B40] Choi B. Jo S. (2013). A low-cost EEG system-based hybrid brain-computer interface for humanoid robot navigation and rECOGnition. PLoS ONE 8:e74583. 10.1371/journal.pone.0074583 24023953 PMC3762758

[B41] Clausen J. (2011). Conceptual and ethical issues with brain-hardware interfaces. Curr. Opin. Psychiatry 24, 495–501. 10.1097/YCO.0b013e32834bb8ca 21934620

[B42] Combaz A. Van Hulle M. M. (2015). Simultaneous detection of p300 and steady-state visually evoked potentials for hybrid brain-computer interface. PLoS ONE 10:e0121481. 10.1371/journal.pone.0121481 25815815 PMC4376875

[B43] Congedo M. Barachant A. Andreev A. (2013). A new generation of brain-computer interface based on riemannian geometry. arXiv preprint arXiv:1310.8115.

[B44] Congedo M. Barachant A. Bhatia R. (2017). Riemannian geometry for EEG-based brain-computer interfaces; a primer and a review. Brain Comput. Interfaces 4, 155–174. 10.1080/2326263X.2017.1297192

[B45] Corsi M.-C. Chavez M. Schwartz D. Hugueville L. Khambhati A. N. Bassett D. S.. (2019). Integrating EEG and MEG signals to improve motor imagery classification in brain-computer interface. Int. J. Neural Syst. 29:1850014. 10.1142/S0129065718500144 29768971

[B46] da Silva F. L. (2013). EEG and MEG: relevance to neuroscience. Neuron 80, 1112–1128. 10.1016/j.neuron.2013.10.017 24314724

[B47] Darvishi S. Gharabaghi A. Boulay C. B. Ridding M. C. Abbott D. Baumert M. (2017). Proprioceptive feedback facilitates motor imagery-related operant learning of sensorimotor β-band modulation. Front. Neurosci. 11:60. 10.3389/fnins.2017.00060 28232788 PMC5299002

[B48] De Marchis C. Monteiro T. S. Simon-Martinez C. Conforto S. Gharabaghi A. (2016). Multi-contact functional electrical stimulation for hand opening: electrophysiologically driven identification of the optimal stimulation site. J. Neuroeng. Rehabil. 13, 1–9. 10.1186/s12984-016-0129-6 26955873 PMC4782521

[B49] de Negueruela C. Broschart M. Menon C. Millán J. d. R. (2011). Brain-computer interfaces for space applications. Pers. Ubiquit. Comput. 15, 527–537. 10.1007/s00779-010-0322-8

[B50] Debener S. Ullsperger M. Siegel M. Engel A. K. (2006). Single-trial EEG-fMRI reveals the dynamics of cognitive function. Trends Cogn. Sci. 10, 558–563. 10.1016/j.tics.2006.09.010 17074530

[B51] Deisseroth K. Schnitzer M. J. (2013). Engineering approaches to illuminating brain structure and dynamics. Neuron 80, 568–577. 10.1016/j.neuron.2013.10.032 24183010 PMC5731466

[B52] Deng X. Yu Z. L. Lin C. Gu Z. Li Y. (2019). A bayesian shared control approach for wheelchair robot with brain machine interface. IEEE Trans. Neural Syst. Rehabil. Eng. 28, 328–338. 10.1109/TNSRE.2019.2958076 31825869

[B53] Denning T. Matsuoka Y. Kohno T. (2009). Neurosecurity: security and privacy for neural devices. Neurosurg. Focus 27:E7. 10.3171/2009.4.FOCUS0985 19569895

[B54] Di Flumeri G. Aricó P. Borghini G. Sciaraffa N. Di Florio A. Babiloni F. (2019). The dry revolution: evaluation of three different EEG dry electrode types in terms of signal spectral features, mental states classification and usability. Sensors 19:1365. 10.3390/s19061365 30893791 PMC6470960

[B55] Dobkin B. H. (2007). Brain-computer interface technology as a tool to augment plasticity and outcomes for neurological rehabilitation. J. Physiol. 579, 637–642. 10.1113/jphysiol.2006.123067 17095557 PMC2151380

[B56] Donoghue J. P. (2008). Bridging the brain to the world: a perspective on neural interface systems. Neuron 60, 511–521. 10.1016/j.neuron.2008.10.037 18995827

[B57] Downey J. E. Weiss J. M. Muelling K. Venkatraman A. Valois J.-S. Hebert M.. (2016). Blending of brain-machine interface and vision-guided autonomous robotics improves neuroprosthetic arm performance during grasping. J. Neuroeng. Rehabil. 13, 1–12. 10.1186/s12984-016-0134-9 26987662 PMC4797113

[B58] Eaton M. L. Illes J. (2007). Commercializing cognitive neurotechnology–the ethical terrain. Nat. Biotechnol. 25, 393–397. 10.1038/nbt0407-393 17420741

[B59] Edelman B. J. Baxter B. He B. (2015). EEG source imaging enhances the decoding of complex right-hand motor imagery tasks. IEEE Trans. Biomed. Eng. 63, 4–14. 10.1109/TBME.2015.2467312 26276986 PMC4716869

[B60] Faress A. Chau T. (2013). Towards a multimodal brain-computer interface: combining fNIRS and ftcd measurements to enable higher classification accuracy. Neuroimage 77, 186–194. 10.1016/j.neuroimage.2013.03.028 23541802

[B61] Farwell L. A. Donchin E. (1988). Talking off the top of your head: toward a mental prosthesis utilizing event-related brain potentials. Electroencephalogr. Clin. Neurophysiol. 70, 510–523. 10.1016/0013-4694(88)90149-6 2461285

[B62] Farwell L. A. Richardson D. C. Richardson G. M. Furedy J. J. (2014). Brain fingerprinting classification concealed information test detects us navy military medical information with p300. Front. Neurosci. 8:410. 10.3389/fnins.2014.00410 25565941 PMC4274905

[B63] Fazli S. Mehnert J. Steinbrink J. Curio G. Villringer A. Müller K.-R.. (2012). Enhanced performance by a hybrid NIRS-EEG brain computer interface. Neuroimage 59, 519–529. 10.1016/j.neuroimage.2011.07.084 21840399

[B64] Filiz E. Arslan R. B. (2020). “Design and implementation of steady state visual evoked potential based brain computer interface video game,” in 2020 IEEE 20th Mediterranean Electrotechnical Conference (MELECON) (Palermo: IEEE), 335–338. 10.1109/MELECON48756.2020.9140710

[B65] Friehs G. Penn R. D. Park M. C. Goldman M. Zerris V. A. Hochberg L. R.. (2006). Initial surgical experience with an intracortical microelectrode array for brain-computer interface applications: 881. Neurosurgery 59:481. 10.1227/00006123-200608000-00119

[B66] Fukuma R. Yanagisawa T. Saitoh Y. Hosomi K. Kishima H. Shimizu T.. (2016). Real-time control of a neuroprosthetic hand by magnetoencephalographic signals from paralysed patients. Sci. Rep. 6, 1–14. 10.1038/srep21781 26904967 PMC4764841

[B67] Galán F. Nuttin M. Lew E. Ferrez P. W. Vanacker G. Philips J.. (2008). A brain-actuated wheelchair: asynchronous and non-invasive brain-computer interfaces for continuous control of robots. Clin. Neurophysiol. 119, 2159–2169. 10.1016/j.clinph.2008.06.001 18621580

[B68] Gandhi V. Prasad G. Coyle D. Behera L. McGinnity T. M. (2014). EEG-based mobile robot control through an adaptive brain-robot interface. IEEE Trans. Syst. Man Cybernet. Syst. 44, 1278–1285. 10.1109/TSMC.2014.2313317

[B69] Gao Z. Wang X. Yang Y. Mu C. Cai Q. Dang W.. (2019). EEG-based spatio-temporal convolutional neural network for driver fatigue evaluation. IEEE Trans. Neural Netw. Learn. Syst. 30, 2755–2763. 10.1109/TNNLS.2018.2886414 30640634

[B70] Ge S. Yang Q. Wang R. Lin P. Gao J. Leng Y.. (2017). A brain-computer interface based on a few-channel EEG-fNIRS bimodal system. IEEE Access 5, 208–218. 10.1109/ACCESS.2016.2637409

[B71] Gilbert F. (2015). Self-estrangement and deep brain stimulation: ethical issues related to forced explantation. Neuroethics 8, 107–114. 10.1007/s12152-014-9224-1

[B72] Gilja V. Chestek C. A. Diester I. Henderson J. M. Deisseroth K. Shenoy K. V. (2011). Challenges and opportunities for next-generation intracortically based neural prostheses. IEEE Trans. Biomed. Eng. 58, 1891–1899. 10.1109/TBME.2011.2107553 21257365 PMC3150197

[B73] Goering S. Yuste R. (2016). On the necessity of ethical guidelines for novel neurotechnologies. Cell 167, 882–885. 10.1016/j.cell.2016.10.029 27814514

[B74] Gonçalves S. I. De Munck J. C. Pouwels P. J. Schoonhoven R. Kuijer J. P. Maurits N. M.. (2006). Correlating the alpha rhythm to bold using simultaneous EEG/fMRI: inter-subject variability. Neuroimage 30, 203–213. 10.1016/j.neuroimage.2005.09.062 16290018

[B75] Grau C. Ginhoux R. Riera A. Nguyen T. L. Chauvat H. Berg M.. (2014). Conscious brain-to-brain communication in humans using non-invasive technologies. PLoS ONE 9:e105225. 10.1371/journal.pone.0105225 25137064 PMC4138179

[B76] Grienberger C. Konnerth A. (2012). Imaging calcium in neurons. Neuron 73, 862–885. 10.1016/j.neuron.2012.02.011 22405199

[B77] Grillner S. Ip N. Koch C. Koroshetz W. Okano H. Polachek M.. (2016). Worldwide initiatives to advance brain research. Nat. Neurosci. 19, 1118–1122. 10.1038/nn.4371 27571190 PMC6047900

[B78] Grosse-Wentrup M. Mattia D. Oweiss K. (2011). Using brain-computer interfaces to induce neural plasticity and restore function. J. Neural Eng. 8:025004. 10.1088/1741-2560/8/2/025004 21436534 PMC4515347

[B79] Guy V. Soriani M.-H. Bruno M. Papadopoulo T. Desnuelle C. Clerc M. (2018). Brain computer interface with the p300 speller: usability for disabled people with amyotrophic lateral sclerosis. Ann. Phys. Rehabil. Med. 61, 5–11. 10.1016/j.rehab.2017.09.004 29024794

[B80] Halder S. Leinfelder T. Schulz S. M. Kübler A. (2019). Neural mechanisms of training an auditory event-related potential task in a brain-computer interface context. Hum. Brain Mapp. 40, 2399–2412. 10.1002/hbm.24531 30693612 PMC6865430

[B81] Hammer E. M. Halder S. Blankertz B. Sannelli C. Dickhaus T. Kleih S.. (2012). Psychological predictors of SMR-BCI performance. Biol. Psychol. 89, 80–86. 10.1016/j.biopsycho.2011.09.006 21964375

[B82] Han C.-H. Müller K.-R. Hwang H.-J. (2020). Brain-switches for asynchronous brain-computer interfaces: a systematic review. Electronics 9:422. 10.3390/electronics9030422

[B83] Hasson U. Nir Y. Levy I. Fuhrmann G. Malach R. (2004). Intersubject synchronization of cortical activity during natural vision. Science 303, 1634–1640. 10.1126/science.1089506 15016991

[B84] He B. Yang L. Wilke C. Yuan H. (2011). Electrophysiological imaging of brain activity and connectivity-challenges and opportunities. IEEE Trans. Biomed. Eng. 58, 1918–1931. 10.1109/TBME.2011.2139210 21478071 PMC3241716

[B85] He H. Wu D. (2019). Transfer learning for brain-computer interfaces: a Euclidean space data alignment approach. IEEE Trans. Biomed. Eng. 67, 399–410. 10.1109/TBME.2019.2913914 31034407

[B86] He H. Wu D. (2020). Different set domain adaptation for brain-computer interfaces: a label alignment approach. IEEE Trans. Neural Syst. Rehabil. Eng.28, 1091–1108. 10.1109/TNSRE.2020.2980299 32167903

[B87] Hinrichs H. Scholz M. Baum A. K. Kam J. W. Knight R. T. Heinze H.-J. (2020). Comparison between a wireless dry electrode EEG system with a conventional wired wet electrode EEG system for clinical applications. Sci. Rep. 10, 1–14. 10.1038/s41598-020-62154-0 32251333 PMC7090045

[B88] Hobson E. V. Fazal S. Shaw P. J. McDermott C. J. (2017). “Anything that makes life's journey better.” Exploring the use of digital technology by people living with motor neurone disease. Amyotrophic Lateral Sclerosis Frontotemporal Degeneration 18, 378–387. 10.1080/21678421.2017.1288253 28631958

[B89] Hochberg L. R. (2013). Intracortical brain-computer interfaces for the restoration of communication and mobility. Biophys. J. 104:376. 10.1016/j.bpj.2012.11.2094

[B90] Honey C. J. Sporns O. Cammoun L. Gigandet X. Thiran J.-P. Meuli R.. (2009). Predicting human resting-state functional connectivity from structural connectivity. Proc. Natl. Acad. Sci. U.S.A. 106, 2035–2040. 10.1073/pnas.0811168106 19188601 PMC2634800

[B91] Honey C. J. Thivierge J.-P. Sporns O. (2010). Can structure predict function in the human brain? Neuroimage 52, 766–776. 10.1016/j.neuroimage.2010.01.071 20116438

[B92] Hong X. Lu Z. K. Teh I. Nasrallah F. A. Teo W. P. Ang K. K.. (2017). Brain plasticity following MI-BCI training combined with tdcs in a randomized trial in chronic subcortical stroke subjects: a preliminary study. Sci. Rep. 7, 1–12. 10.1038/s41598-017-08928-5 28835651 PMC5569072

[B93] Horki P. Solis-Escalante T. Neuper C. Müller-Putz G. (2011). Combined motor imagery and ssvep based BCI control of a 2 DOF artificial upper limb. Med. Biol. Eng. Comput. 49, 567–577. 10.1007/s11517-011-0750-2 21394652

[B94] Huang H. Xie Q. Pan J. He Y. Wen Z. Yu R.. (2019). An EEG-based brain computer interface for emotion rECOGnition and its application in patients with disorder of consciousness. IEEE Trans. Affect. Comput. 10.1109/TAFFC.2019.2901456

[B95] Ienca M. Haselager P. (2016). Hacking the brain: brain-computer interfacing technology and the ethics of neurosecurity. Ethics Inform. Technol. 18, 117–129. 10.1007/s10676-016-9398-9

[B96] Illes J. Bird S. J. (2006). Neuroethics: a modern context for ethics in neuroscience. Trends Neurosci. 29, 511–517. 10.1016/j.tins.2006.07.002 16859760 PMC1656950

[B97] Iturrate I. Antelis J. M. Kubler A. Minguez J. (2009). A noninvasive brain-actuated wheelchair based on a p300 neurophysiological protocol and automated navigation. IEEE Trans. Robot. 25, 614–627. 10.1109/TRO.2009.2020347

[B98] Jayaram V. Alamgir M. Altun Y. Scholkopf B. Grosse-Wentrup M. (2016). Transfer learning in brain-computer interfaces. IEEE Comput. Intell. Mag. 11, 20–31. 10.1109/MCI.2015.2501545

[B99] Jebari K. (2013). Brain machine interface and human enhancement-an ethical review. Neuroethics 6, 617–625. 10.1007/s12152-012-9176-2

[B100] Jensen O. Bahramisharif A. Oostenveld R. Klanke S. Hadjipapas A. Okazaki Y. O.. (2011). Using brain-computer interfaces and brain-state dependent stimulation as tools in cognitive neuroscience. Front. Psychol. 2:100. 10.3389/fpsyg.2011.00100 21687463 PMC3108578

[B101] Jiang L. Stocco A. Losey D. M. Abernethy J. A. Prat C. S. Rao R. P. (2019). Brainnet: a multi-person brain-to-brain interface for direct collaboration between brains. Sci. Rep. 9, 1–11. 10.1038/s41598-019-41895-7 30992474 PMC6467884

[B102] Jin J. Chen Z. Xu R. Miao Y. yu Wang X. Jung T.-P. (2020). Developing a novel tactile p300 brain-computer interface with a cheeks-stim paradigm. IEEE Trans. Biomed. Eng. 67, 2585–2593. 10.1109/TBME.2020.2965178 31940515

[B103] Johnson N. Carey J. Edelman B. Doud A. Grande A. Lakshminarayan K.. (2018). Combined rtms and virtual reality brain-computer interface training for motor recovery after stroke. J. Neural Eng. 15:016009. 10.1088/1741-2552/aa8ce3 28914232 PMC5821060

[B104] Jones S. R. Sliva D. D. (2020). Is alpha asymmetry a byproduct or cause of spatial attention? New evidence alpha neurofeedback controls measures of spatial attention. Neuron 105, 404–406. 10.1016/j.neuron.2019.12.033 32027830 PMC7565086

[B105] Jotterand F. Giordano J. (2011). Transcranial magnetic stimulation, deep brain stimulation and personal identity: ethical questions, and neuroethical approaches for medical practice. Int. Rev. Psychiatry 23, 476–485. 10.3109/09540261.2011.616189 22200137

[B106] Juliano J. M. Spicer R. P. Vourvopoulos A. Lefebvre S. Jann K. Ard T.. (2020). Embodiment is related to better performance on a brain-computer interface in immersive virtual reality: a pilot study. Sensors 20:1204. 10.3390/s20041204 32098317 PMC7070491

[B107] Kaas A. L. Goebel R. Valente G. Sorger B. (2019). Topographic somatosensory imagery for real-time fMRI brain-computer interfacing. Front. Hum. Neurosci. 13:427. 10.3389/fnhum.2019.00427 31920588 PMC6915074

[B108] Kaiju T. Yokota M. Watanabe K. Inoue M. Ando H. Takahashi K.. (2017). High spatiotemporal resolution ECOG recording of somatosensory evoked potentials with flexible micro-electrode arrays. Front. Neural Circ. 11:20. 10.3389/fncir.2017.00020 28442997 PMC5386975

[B109] Kamousi B. Liu Z. He B. (2005). Classification of motor imagery tasks for brain-computer interface applications by means of two equivalent dipoles analysis. IEEE Trans. Neural Syst. Rehabil. Eng. 13, 166–171. 10.1109/TNSRE.2005.847386 16003895

[B110] Kasahara K. DaSalla C. S. Honda M. Hanakawa T. (2015). Neuroanatomical correlates of brain-computer interface performance. Neuroimage 110, 95–100. 10.1016/j.neuroimage.2015.01.055 25659465

[B111] Käthner I. Wriessnegger S. C. Müller-Putz G. R. Kübler A. Halder S. (2014). Effects of mental workload and fatigue on the p300, alpha and theta band power during operation of an ERP (p300) brain-computer interface. Biol. Psychol. 102, 118–129. 10.1016/j.biopsycho.2014.07.014 25088378

[B112] Kaufmann T. Vögele C. Sütterlin S. Lukito S. Kübler A. (2012). Effects of resting heart rate variability on performance in the p300 brain-computer interface. Int. J. Psychophysiol. 83, 336–341. 10.1016/j.ijpsycho.2011.11.018 22172335

[B113] Kauhanen L. Nykopp T. Lehtonen J. Jylanki P. Heikkonen J. Rantanen P.. (2006). EEG and MEG brain-computer interface for tetraplegic patients. IEEE Trans. Neural Syst. Rehabil. Eng. 14, 190–193. 10.1109/TNSRE.2006.875546 16792291

[B114] Keefer E. W. Botterman B. R. Romero M. I. Rossi A. F. Gross G. W. (2008). Carbon nanotube coating improves neuronal recordings. Nat. Nanotechnol. 3, 434–439. 10.1038/nnano.2008.174 18654569

[B115] Khalaf A. Sejdic E. Akcakaya M. (2019). A novel motor imagery hybrid brain computer interface using EEG and functional transcranial doppler ultrasound. J. Neurosci. Methods 313, 44–53. 10.1016/j.jneumeth.2018.11.017 30590086

[B116] Khodagholy D. Doublet T. Quilichini P. Gurfinkel M. Leleux P. Ghestem A.. (2013). *In vivo* recordings of brain activity using organic transistors. Nat. Commun. 4, 1–7. 10.1038/ncomms2573 23481383 PMC3615373

[B117] Kleih S. C. Kübler A. (2013). Empathy, motivation, and p300 BCI performance. Front. Hum. Neurosci. 7:642. 10.3389/fnhum.2013.00642 24146640 PMC3797970

[B118] Kleinschmidt A. Sterzer P. Rees G. (2012). Variability of perceptual multistability: from brain state to individual trait. Philos. Trans. R. Soc. B Biol. Sci. 367, 988–1000. 10.1098/rstb.2011.0367 22371620 PMC3282312

[B119] Krusienski D. J. Grosse-Wentrup M. Galán F. Coyle D. Miller K. J. Forney E.. (2011). Critical issues in state-of-the-art brain-computer interface signal processing. J. Neural Eng. 8:025002. 10.1088/1741-2560/8/2/025002 21436519 PMC3412170

[B120] Kruskal P. B. Jiang Z. Gao T. Lieber C. M. (2015). Beyond the patch clamp: nanotechnologies for intracellular recording. Neuron 86, 21–24. 10.1016/j.neuron.2015.01.004 25856481

[B121] Kwon O.-Y. Lee M.-H. Guan C. Lee S.-W. (2019). Subject-independent brain-computer interfaces based on deep convolutional neural networks. IEEE Trans. Neural Netw. Learn. Syst. 31, 3839–3852. 10.1109/TNNLS.2019.2946869 31725394

[B122] LaFleur K. Cassady K. Doud A. Shades K. Rogin E. He B. (2013). Quadcopter control in three-dimensional space using a noninvasive motor imagery-based brain-computer interface. J. Neural Eng. 10:046003. 10.1088/1741-2560/10/4/046003 23735712 PMC3839680

[B123] Lajoie G. Krouchev N. I. Kalaska J. F. Fairhall A. L. Fetz E. E. (2017). Correlation-based model of artificially induced plasticity in motor cortex by a bidirectional brain-computer interface. PLoS Comput. Biol. 13:e1005343. 10.1371/journal.pcbi.1005343 28151957 PMC5313237

[B124] Leamy D. J. Kocijan J. Domijan K. Duffin J. Roche R. A. Commins S.. (2014). An exploration of EEG features during recovery following stroke-implications for BCI-mediated neurorehabilitation therapy. J. Neuroeng. Rehabil. 11:9. 10.1186/1743-0003-11-9 24468185 PMC3996183

[B125] Lebedev M. A. Nicolelis M. A. (2017). Brain-machine interfaces: from basic science to neuroprostheses and neurorehabilitation. Physiol. Rev. 97, 767–837. 10.1152/physrev.00027.2016 28275048

[B126] Lécuyer A. Lotte F. Reilly R. B. Leeb R. Hirose M. Slater M. (2008). Brain-computer interfaces, virtual reality, and videogames. Computer 41, 66–72. 10.1109/MC.2008.410

[B127] Liao L.-D. Lin C.-T. McDowell K. Wickenden A. E. Gramann K. Jung T.-P.. (2012). Biosensor technologies for augmented brain-computer interfaces in the next decades. Proc. IEEE 100, 1553–1566. 10.1109/JPROC.2012.2184829

[B128] Lin C.-T. Chen Y.-C. Huang T.-Y. Chiu T.-T. Ko L.-W. Liang S.-F.. (2008). Development of wireless brain computer interface with embedded multitask scheduling and its application on real-time driver's drowsiness detection and warning. IEEE Trans. Biomed. Eng. 55, 1582–1591. 10.1109/TBME.2008.918566 18440904

[B129] Lina J.-M. Chowdhury R. Lemay E. Kobayashi E. Grova C. (2012). Wavelet-based localization of oscillatory sources from magnetoencephalography data. IEEE Trans. Biomed. Eng. 61, 2350–2364. 10.1109/TBME.2012.2189883 22410322

[B130] Lotte F. Bougrain L. Cichocki A. Clerc M. Congedo M. Rakotomamonjy A.. (2018). A review of classification algorithms for EEG-based brain-computer interfaces: a 10 year update. J. Neural Eng. 15:031005. 10.1088/1741-2552/aab2f2 29488902

[B131] Lotte F. Congedo M. Lécuyer A. Lamarche F. Arnaldi B. (2007). A review of classification algorithms for EEG-based brain-computer interfaces. J. Neural Eng. 4:R1. 10.1088/1741-2560/4/2/R01 17409472

[B132] Lotte F. Guan C. (2010). Regularizing common spatial patterns to improve BCI designs: unified theory and new algorithms. IEEE Trans. Biomed. Eng. 58, 355–362. 10.1109/TBME.2010.2082539 20889426

[B133] Lu J. Mamun K. A. Chau T. (2015). Pattern classification to optimize the performance of transcranial doppler ultrasonography-based brain machine interface. Pattern Recogn. Lett. 66, 135–143. 10.1016/j.patrec.2015.07.020

[B134] Luu T. P. He Y. Brown S. Nakagome S. Contreras-Vidal J. L. (2016). Gait adaptation to visual kinematic perturbations using a real-time closed-loop brain-computer interface to a virtual reality avatar. J. Neural Eng. 13:036006. 10.1088/1741-2560/13/3/036006 27064824 PMC5726869

[B135] Lystad R. P. Pollard H. (2009). Functional neuroimaging: a brief overview and feasibility for use in chiropractic research. J. Can. Chiropract. Assoc. 53:59. 19421353 PMC2652631

[B136] Mahjoory K. Nikulin V. V. Botrel L. Linkenkaer-Hansen K. Fato M. M. Haufe S. (2017). Consistency of EEG source localization and connectivity estimates. Neuroimage 152, 590–601. 10.1016/j.neuroimage.2017.02.076 28300640

[B137] Mak J. N. Wolpaw J. R. (2009). Clinical applications of brain-computer interfaces: current state and future prospects. IEEE Rev. Biomed. Eng. 2, 187–199. 10.1109/RBME.2009.2035356 20442804 PMC2862632

[B138] Mantini D. Perrucci M. G. Del Gratta C. Romani G. L. Corbetta M. (2007). Electrophysiological signatures of resting state networks in the human brain. Proc. Natl. Acad. Sci. U.S.A. 104, 13170–13175. 10.1073/pnas.0700668104 17670949 PMC1941820

[B139] Marathe A. R. Lawhern V. J. Wu D. Slayback D. Lance B. J. (2015). Improved neural signal classification in a rapid serial visual presentation task using active learning. IEEE Trans. Neural Syst. Rehabil. Eng. 24, 333–343. 10.1109/TNSRE.2015.2502323 26600162

[B140] Marchesotti S. Bassolino M. Serino A. Bleuler H. Blanke O. (2016). Quantifying the role of motor imagery in brain-machine interfaces. Sci. Rep. 6:24076. 10.1038/srep24076 27052520 PMC4823701

[B141] Marchesotti S. Martuzzi R. Schurger A. Blefari M. L. del Millán J. R. Bleuler H.. (2017). Cortical and subcortical mechanisms of brain-machine interfaces. Hum. Brain Mapp. 38, 2971–2989. 10.1002/hbm.23566 28321973 PMC6866852

[B142] Marini F. Lee C. Wagner J. Makeig S. Gola M. (2019). A comparative evaluation of signal quality between a research-grade and a wireless dry-electrode mobile EEG system. J. Neural Eng. 16:054001. 10.1088/1741-2552/ab21f2 31096191

[B143] Martin M. V. Cho V. Aversano G. (2016). Detection of subconscious face rECOGnition using consumer-grade brain-computer interfaces. ACM Trans. Appl. Percept. 14, 1–20. 10.1145/2955097

[B144] Maslen H. Pugh J. Savulescu J. (2015). The ethics of deep brain stimulation for the treatment of anorexia nervosa. Neuroethics 8, 215–230. 10.1007/s12152-015-9240-9 26594256 PMC4643100

[B145] Matthews F. Pearlmutter B. A. Wards T. E. Soraghan C. Markham C. (2007). Hemodynamics for brain-computer interfaces. IEEE Signal Process. Mag. 25, 87–94. 10.1109/MSP.2008.4408445

[B146] McCane L. M. Heckman S. M. McFarland D. J. Townsend G. Mak J. N. Sellers E. W.. (2015). P300-based brain-computer interface (BCI) event-related potentials (ERPs): people with amyotrophic lateral sclerosis (ALS) vs. age-matched controls. Clin. Neurophysiol. 126, 2124–2131. 10.1016/j.clinph.2015.01.013 25703940 PMC4529383

[B147] McClinton W. Garcia S. Andujar M. (2019). “An immersive brain painting: the effects of brain painting in a virtual reality environment,” in International Conference on Human-Computer Interaction (Copenhagen: Springer), 436–445. 10.1007/978-3-030-22419-6_31

[B148] McCullagh P. Lightbody G. Zygierewicz J. Kernohan W. G. (2014). Ethical challenges associated with the development and deployment of brain computer interface technology. Neuroethics 7, 109–122. 10.1007/s12152-013-9188-6

[B149] McFarland D. J. Anderson C. W. Muller K.-R. Schlogl A. Krusienski D. J. (2006). Bci meeting 2005-workshop on BCI signal processing: feature extraction and translation. IEEE Trans. Neural Syst. Rehabil. Eng. 14, 135–138. 10.1109/TNSRE.2006.875637 16792278

[B150] McMillan G. R. Calhoun G. Middendorf M. Schnurer J. Ingle D. Nasman V. (1995). “Direct brain interface utilizing self-regulation of steady-state visual evoked response (SSVER),” in Proc. RESNA 95 Annual Conf . (Vancouver, BC), 693–695.

[B151] Megan T. D. Cohen J. D. Lee R. F. Norman K. A. Turk-Browne N. B. (2015). Closed-loop training of attention with real-time brain imaging. Nat. Neurosci. 18, 470–475. 10.1038/nn.3940 25664913 PMC4503600

[B152] Mellinger J. Schalk G. Braun C. Preissl H. Rosenstiel W. Birbaumer N.. (2007). An MEG-based brain-computer interface (BCI). Neuroimage 36, 581–593. 10.1016/j.neuroimage.2007.03.019 17475511 PMC2017111

[B153] Menon C. De Negueruela C. Millán J. d. R. Tonet O. Carpi F. Broschart M.. (2009). Prospects of brain-machine interfaces for space system control. Acta Astronaut. 64, 448–456. 10.1016/j.actaastro.2008.09.008

[B154] Mestais C. S. Charvet G. Sauter-Starace F. Foerster M. Ratel D. Benabid A. L. (2014). Wimagine: wireless 64-channel ECOG recording implant for long term clinical applications. IEEE Trans. Neural Syst. Rehabil. Eng. 23, 10–21. 10.1109/TNSRE.2014.2333541 25014960

[B155] Milan J. D. R. Carmena J. M. (2010). Invasive or noninvasive: understanding brain-machine interface technology [conversations in BME]. IEEE Eng. Med. Biol. Mag. 29, 16–22. 10.1109/MEMB.2009.935475 20209672

[B156] Millan J. R. Renkens F. Mourino J. Gerstner W. (2004). Noninvasive brain-actuated control of a mobile robot by human EEG. IEEE Trans. Biomed. Eng. 51, 1026–1033. 10.1109/TBME.2004.827086 15188874

[B157] Min B.-K. Hämäläinen M. S. Pantazis D. (2020). New cognitive neurotechnology facilitates studies of cortical-subcortical interactions. Trends Biotechnol. 38, 952–962. 10.1016/j.tibtech.2020.03.003 32278504 PMC7442676

[B158] Min B.-K. Marzelli M. J. Yoo S.-S. (2010). Neuroimaging-based approaches in the brain-computer interface. Trends Biotechnol. 28, 552–560. 10.1016/j.tibtech.2010.08.002 20810180

[B159] Mota A. R. Duarte L. Rodrigues D. Martins A. Machado A. Vaz F.. (2013). Development of a quasi-dry electrode for EEG recording. Sensors Actuat A Phys. 199, 310–317. 10.1016/j.sna.2013.06.013

[B160] Mudgal S. K. Sharma S. S. K. Chaturvedi I. Sharma A. (2020). Brain computer interface advancement in neurosciences: applications and issues. Interdiscipl. Neurosurg. 20:100694. 10.1016/j.inat.2020.100694

[B161] Müller J. L. Röder C. H. Schuierer G. Klein H. (2002). Motor-induced brain activation in cortical, subcortical and cerebellar regions in schizophrenic inpatients. A whole brain fMRI fingertapping study. Prog. Neuropsychopharmacol. Biol. Psychiatry 26, 421–426. 10.1016/S0278-5846(01)00271-8 11999890

[B162] Muller-Putz G. R. Scherer R. Neuper C. Pfurtscheller G. (2006). Steady-state somatosensory evoked potentials: suitable brain signals for brain-computer interfaces? IEEE Trans. Neural Syst. Rehabil. Eng. 14, 30–37. 10.1109/TNSRE.2005.863842 16562629

[B163] Murovec N. Heilinger A. Xu R. Ortner R. Spataro R. La Bella V.. (2020). Effects of a vibro-tactile p300 based brain-computer interface on the coma recovery scale-revised in patients with disorders of consciousness. Front. Neurosci. 14:294. 10.3389/fnins.2020.00294 32327970 PMC7161577

[B164] Nagel S. Spüler M. (2019). World's fastest brain-computer interface: combining EEG2code with deep learning. PLoS ONE 14:e0221909. 10.1371/journal.pone.0221909 31490999 PMC6730910

[B165] Nakazawa E. Yamamoto K. Tachibana K. Toda S. Takimoto Y. Akabayashi A. (2016). Ethics of decoded neurofeedback in clinical research, treatment, and moral enhancement. AJOB Neurosci. 7, 110–117. 10.1080/21507740.2016.1172134

[B166] Naseer N. Hong K.-S. (2015). fNIRS-based brain-computer interfaces: a review. Front. Hum. Neurosci. 9:3. 10.3389/fnhum.2015.00003 25674060 PMC4309034

[B167] Nicolas-Alonso L. F. Gomez-Gil J. (2012). Brain computer interfaces, a review. Sensors 12, 1211–1279. 10.3390/s120201211 22438708 PMC3304110

[B168] Nicolelis M. A. Lebedev M. A. (2009). Principles of neural ensemble physiology underlying the operation of brain-machine interfaces. Nat. Rev. Neurosci. 10, 530–540. 10.1038/nrn2653 19543222

[B169] Nijboer F. Birbaumer N. Kubler A. (2010). The influence of psychological state and motivation on brain-computer interface performance in patients with amyotrophic lateral sclerosis-A longitudinal study. Front. Neurosci. 4:55. 10.3389/fnins.2010.00055 20700521 PMC2916671

[B170] Nijholt A. Poel M. (2016). “Multi-brain BCI: Characteristics and social interactions,” in International Conference on Augmented Cognition (Las Vegas, NV: Springer), 79–90. 10.1007/978-3-319-39955-3_8

[B171] Norton J. J. Lee D. S. Lee J. W. Lee W. Kwon O. Won P.. (2015). Soft, curved electrode systems capable of integration on the auricle as a persistent brain-computer interface. Proc. Natl. Acad. Sci. U.S.A. 112, 3920–3925. 10.1073/pnas.1424875112 25775550 PMC4386388

[B172] Orsborn A. L. Moorman H. G. Overduin S. A. Shanechi M. M. Dimitrov D. F. Carmena J. M. (2014). Closed-loop decoder adaptation shapes neural plasticity for skillful neuroprosthetic control. Neuron 82, 1380–1393. 10.1016/j.neuron.2014.04.048 24945777

[B173] Ortiz F. González J. Montes A. González N. Peña A. (2016). Induction of emotional states in people with disabilities through film clips using brain computer interfaces. IEEE Latin Am. Trans. 14, 563–568. 10.1109/TLA.2016.7437193

[B174] Oxley T. J. Opie N. L. John S. E. Rind G. S. Ronayne S. M. Burkitt A. N.. (2017). “A minimally invasive endovascular stent-electrode array for chronic recordings of cortical neural activity,” in Brain-Computer Interface Research, eds Guger C. Allison B. Lebedev M. (Cham: Springer), 55–63. 10.1007/978-3-319-64373-1_6

[B175] Oxley T. J. Opie N. L. John S. E. Rind G. S. Ronayne S. M. Wheeler T. L.. (2016). Minimally invasive endovascular stent-electrode array for high-fidelity, chronic recordings of cortical neural activity. Nat. Biotechnol. 34, 320–327. 10.1038/nbt.3428 26854476

[B176] Oxley T. J. Yoo P. E. Rind G. S. Ronayne S. M. Lee C. S. Bird C.. (2020). Motor neuroprosthesis implanted with neurointerventional surgery improves capacity for activities of daily living tasks in severe paralysis: first in-human experience. J. NeuroIntervent. Surg. 10.1136/neurintsurg-2020-016862 33115813 PMC7848062

[B177] Pahwa M. Kusner M. Hacker C. D. Bundy D. T. Weinberger K. Q. Leuthardt E. C. (2015). Optimizing the detection of wakeful and sleep-like states for future electrocorticographic brain computer interface applications. PLoS ONE 10:e0142947. 10.1371/journal.pone.0142947 26562013 PMC4643046

[B178] Pais-Vieira M. Lebedev M. Kunicki C. Wang J. Nicolelis M. A. (2013). A brain-to-brain interface for real-time sharing of sensorimotor information. Sci. Rep. 3:1319. 10.1038/srep01319 23448946 PMC3584574

[B179] Pandarinath C. Nuyujukian P. Blabe C. H. Sorice B. L. Saab J. Willett F. R.. (2017). High performance communication by people with paralysis using an intracortical brain-computer interface. eLife 6:e18554. 10.7554/eLife.18554 28220753 PMC5319839

[B180] Park W. Kwon G. H. Kim Y.-H. Lee J.-H. Kim L. (2016). EEG response varies with lesion location in patients with chronic stroke. J. Neuroeng. Rehabil. 13, 1–10. 10.1186/s12984-016-0120-2 26935230 PMC4776402

[B181] Perdikis S. Tonin L. Millan J. d. R. (2017). Brain racers. IEEE Spectrum 54, 44–51. 10.1109/MSPEC.2017.8012239

[B182] Petrov Y. Nador J. Hughes C. Tran S. Yavuzcetin O. Sridhar S. (2014). Ultra-dense EEG sampling results in two-fold increase of functional brain information. Neuroimage 90, 140–145. 10.1016/j.neuroimage.2013.12.041 24398333

[B183] Pfurtscheller G. Guger C. Müller G. Krausz G. Neuper C. (2000). Brain oscillations control hand orthosis in a tetraplegic. Neurosci. Lett. 292, 211–214. 10.1016/S0304-3940(00)01471-3 11018314

[B184] Pfurtscheller G. Solis-Escalante T. Ortner R. Linortner P. Muller-Putz G. R. (2010). Self-paced operation of an ssvep-based orthosis with and without an imagery-based “brain switch:” a feasibility study towards a hybrid BCI. IEEE Trans. Neural Syst. Rehabil. Eng. 18, 409–414. 10.1109/TNSRE.2010.2040837 20144923

[B185] Piastra M. C. Nüßing A. Vorwerk J. Clerc M. Engwer C. Wolters C. H. (2020). A comprehensive study on electroencephalography and magnetoencephalography sensitivity to cortical and subcortical sources. Hum. Brain Mapp. 10.1002/hbm.25272 33156569 PMC7856654

[B186] Picard R. W. (2000). Affective Computing. Cambridge, MA; London: MIT Press. 10.7551/mitpress/1140.001.0001

[B187] Piho L. Tjahjadi T. (2018). A mutual information based adaptive windowing of informative EEG for emotion recognition. IEEE Trans. Affect. Comput. 11, 722–735. 10.1109/TAFFC.2018.2840973

[B188] Putze F. Vourvopoulos A. Lécuyer A. Krusienski D. i Badia S. B. Mullen T.. (2020). Brain-computer interfaces and augmented/virtual reality. Front. Hum. Neurosci. 14:144. 10.3389/fnhum.2020.00144 32477080 PMC7235375

[B189] Qin L. Ding L. He B. (2004). Motor imagery classification by means of source analysis for brain-computer interface applications. J. Neural Eng. 1:135. 10.1088/1741-2560/1/3/002 15876632 PMC1945182

[B190] Ramoser H. Muller-Gerking J. Pfurtscheller G. (2000). Optimal spatial filtering of single trial EEG during imagined hand movement. IEEE Trans. Rehabil. Eng. 8, 441–446. 10.1109/86.895946 11204034

[B191] Ramos-Murguialday A. Broetz D. Rea M. Läer L. Yilmaz Ö. Brasil F. L.. (2013). Brain-machine interface in chronic stroke rehabilitation: a controlled study. Ann. Neurol. 74, 100–108. 10.1002/ana.23879 23494615 PMC3700597

[B192] Rao R. P. Stocco A. Bryan M. Sarma D. Youngquist T. M. Wu J.. (2014). A direct brain-to-brain interface in humans. PLoS ONE 9:e111332. 10.1371/journal.pone.0111332 25372285 PMC4221017

[B193] Rashid M. Sulaiman N. P. P Abdul Majeed A. Musa R. M. Ab Nasir A. F.. (2020). Current status, challenges, and possible solutions of EEG-based brain-computer interface: a comprehensive review. Front. Neurorobot. 14:25. 10.3389/fnbot.2020.00025 32581758 PMC7283463

[B194] Ray A. M. Sitaram R. Rana M. Pasqualotto E. Buyukturkoglu K. Guan C.. (2015). A subject-independent pattern-based brain-computer interface. Front. Behav. Neurosci. 9:269. 10.3389/fnbeh.2015.00269 26539089 PMC4611064

[B195] Resendez S. L. Jennings J. H. Ung R. L. Namboodiri V. M. K. Zhou Z. C. Otis J. M.. (2016). Visualization of cortical, subcortical and deep brain neural circuit dynamics during naturalistic mammalian behavior with head-mounted microscopes and chronically implanted lenses. Nat. Protoc. 11:566. 10.1038/nprot.2016.021 26914316 PMC5239057

[B196] Rezazadeh Sereshkeh A. Yousefi R. Wong A. T. Rudzicz F. Chau T. (2019). Development of a ternary hybrid fNIRS-EEG brain-computer interface based on imagined speech. Brain Comput. Interfaces 6, 128–140. 10.1080/2326263X.2019.1698928

[B197] Romero-Laiseca M. A. Delisle-Rodriguez D. Cardoso V. Gurve D. Loterio F. Nascimento J. H. P.. (2020). A low-cost lower-limb brain-machine interface triggered by pedaling motor imagery for post-stroke patients rehabilitation. IEEE Trans. Neural Syst. Rehabil. Eng. 28, 988–996. 10.1109/TNSRE.2020.2974056 32078552

[B198] Rosenboom D. (2014). Active imaginative listening–A neuromusical critique. Front. Neurosci. 8:251. 10.3389/fnins.2014.00251 25202231 PMC4141212

[B199] Rosenfeld J. V. Wong Y. T. (2017). Neurobionics and the brain-computer interface: current applications and future horizons. Med. J. Australia 206, 363–368. 10.5694/mja16.01011 28446119

[B200] Roth B. L. (2016). Dreadds for neuroscientists. Neuron 89, 683–694. 10.1016/j.neuron.2016.01.040 26889809 PMC4759656

[B201] Saeedi S. Chavarriaga R. Millán J. d. R. (2016). Long-term stable control of motor-imagery BCI by a locked-in user through adaptive assistance. IEEE Trans. Neural Syst. Rehabil. Eng. 25, 380–391. 10.1109/TNSRE.2016.2645681 28055886

[B202] Saha S. Ahmed K. I. Mostafa R. Khandoker A. H. Hadjileontiadis L. (2017). Enhanced inter-subject brain computer interface with associative sensorimotor oscillations. Healthcare Technol. Lett. 4, 39–43. 10.1049/htl.2016.0073 28529762 PMC5435948

[B203] Saha S. Ahmed K. I. U. Mostafa R. Hadjileontiadis L. Khandoker A. (2018). Evidence of variabilities in EEG dynamics during motor imagery-based multiclass brain-computer interface. IEEE Trans. Neural Syst. Rehabil. Eng. 26, 371–382. 10.1109/TNSRE.2017.2778178 29432108

[B204] Saha S. Baumert M. (2020). Intra-and inter-subject variability in EEG-based sensorimotor brain computer interface: a review. Front. Comput. Neurosci. 13:87. 10.3389/fncom.2019.00087 32038208 PMC6985367

[B205] Saha S. Hossain M. Ahmed K. I. U. Mostafa R. Hadjileontiadis L. J. Khandoker A. H.. (2019a). Wavelet entropy-based inter-subject associative cortical source localization for sensorimotor BCI. Front. Neuroinform. 13:47. 10.3389/fninf.2019.00047 31396068 PMC6664070

[B206] Saha S. Mamun K. A. Ahmed K. Mostafa R. Naik G. R. Khandoker A.. (2019b). Progress in brain computer interfaces: challenges and trends. arXiv preprint arXiv:1901.03442. 10.3389/fnsys.2021.578875 PMC7947348 33716680

[B207] Samek W. Meinecke F. C. Müller K.-R. (2013). Transferring subspaces between subjects in brain-computer interfacing. IEEE Trans. Biomed. Eng. 60, 2289–2298. 10.1109/TBME.2013.2253608 23529075

[B208] Sand D. Peremen Z. Haor D. Arkadir D. Bergman H. Geva A. (2017). Optimization of deep brain stimulation in stn among patients with Parkinson's disease using a novel EEG-based tool. Brain Stimulat. 10:510. 10.1016/j.brs.2017.01.490

[B209] Sannelli C. Vidaurre C. Müller K.-R. Blankertz B. (2016). Ensembles of adaptive spatial filters increase BCI performance: an online evaluation. J. Neural Eng. 13:046003. 10.1088/1741-2560/13/4/046003 27187530

[B210] Sauter-Starce F. Ratel D. Cretallaz C. Foerster M. Lambert A. Gaude C.. (2019). Long-term sheep implantation of wimagine ®, a wireless 64-channels electrocorticogram recorder. Front. Neurosci. 13:847. 10.3389/fnins.2019.00847 31496929 PMC6712079

[B211] Schalk G. (2010). Can electrocorticography (ECOG) support robust and powerful brain-computer interfaces? Front. Neuroeng. 3:9. 10.3389/fneng.2010.00009 20631853 PMC2903308

[B212] Schaworonkow N. Triesch J. Ziemann U. Zrenner C. (2019). EEG-triggered tms reveals stronger brain state-dependent modulation of motor evoked potentials at weaker stimulation intensities. Brain Stimulat. 12, 110–118. 10.1016/j.brs.2018.09.009 30268710

[B213] Schicktanz S. Amelung T. Rieger J. W. (2015). Qualitative assessment of patients' attitudes and expectations toward BCIs and implications for future technology development. Front. Syst. Neurosci. 9:64. 10.3389/fnsys.2015.00064 25964745 PMC4410612

[B214] Scholkmann F. Kleiser S. Metz A. J. Zimmermann R. Pavia J. M. Wolf U.. (2014). A review on continuous wave functional near-infrared spectroscopy and imaging instrumentation and methodology. Neuroimage 85, 6–27. 10.1016/j.neuroimage.2013.05.004 23684868

[B215] Schreuder M. Blankertz B. Tangermann M. (2010). A new auditory multi-class brain-computer interface paradigm: spatial hearing as an informative cue. PLoS ONE 5:e9813. 10.1371/journal.pone.0009813 20368976 PMC2848564

[B216] Schwartz A. B. Cui X. T. Weber D. J. Moran D. W. (2006). Brain-controlled interfaces: movement restoration with neural prosthetics. Neuron 52, 205–220. 10.1016/j.neuron.2006.09.019 17015237

[B217] Sekhavat Y. A. (2020). Battle of minds: a new interaction approach in BCI games through competitive reinforcement. Multimedia Tools Appl. 79, 3449–3464. 10.1007/s11042-019-07963-w

[B218] Seo D. Neely R. M. Shen K. Singhal U. Alon E. Rabaey J. M.. (2016). Wireless recording in the peripheral nervous system with ultrasonic neural dust. Neuron 91, 529–539. 10.1016/j.neuron.2016.06.034 27497221

[B219] Shahriari Y. Vaughan T. M. McCane L. Allison B. Z. Wolpaw J. R. Krusienski D. J. (2019). An exploration of BCI performance variations in people with amyotrophic lateral sclerosis using longitudinal EEG data. J. Neural Eng. 16:056031. 10.1088/1741-2552/ab22ea 31108477 PMC7951329

[B220] Shibata K. Watanabe T. Sasaki Y. Kawato M. (2011). Perceptual learning incepted by decoded fMRI neurofeedback without stimulus presentation. Science 334, 1413–1415. 10.1126/science.1212003 22158821 PMC3297423

[B221] Shih J. J. Krusienski D. J. Wolpaw J. R. (2012). “Brain-computer interfaces in medicine,” in Mayo Clinic Proceedings (Elsevier), 268–279. 10.1016/j.mayocp.2011.12.008 PMC3497935 22325364

[B222] Singh A. K. Wang Y.-K. King J.-T. Lin C.-T. (2020). Extended interaction with a BCI video game changes resting-state brain activity. IEEE Trans. Cogn. Dev. Syst. 12, 809–823. 10.1109/TCDS.2020.2985102

[B223] Sitaram R. Ros T. Stoeckel L. Haller S. Scharnowski F. Lewis-Peacock J.. (2017). Closed-loop brain training: the science of neurofeedback. Nat. Rev. Neurosci. 18, 86–100. 10.1038/nrn.2016.164 28003656

[B224] Sitaram R. Weiskopf N. Caria A. Veit R. Erb M. Birbaumer N. (2007). fMRI brain-computer interfaces. IEEE Signal Process. Mag. 25, 95–106. 10.1109/MSP.2008.4408446

[B225] Škola F. Tinková S. Liarokapis F. (2019). Progressive training for motor imagery brain-computer interfaces using gamification and virtual reality embodiment. Front. Hum. Neurosci. 13:329. 10.3389/fnhum.2019.00329 31616269 PMC6775193

[B226] Song M. Kim J. (2019). A paradigm to enhance motor imagery using rubber hand illusion induced by visuo-tactile stimulus. IEEE Trans. Neural Syst. Rehabil. Eng. 27, 477–486. 10.1109/TNSRE.2019.2895029 30703031

[B227] Song T. Zheng W. Song P. Cui Z. (2018). EEG emotion recognition using dynamical graph convolutional neural networks. IEEE Trans. Affect. Comput. 11, 532–541. 10.1109/TAFFC.2018.2817622

[B228] Spataro R. Chella A. Allison B. Giardina M. Sorbello R. Tramonte S.. (2017). Reaching and grasping a glass of water by locked-in als patients through a BCI-controlled humanoid robot. Front. Hum. Neurosci. 11:68. 10.3389/fnhum.2017.00068 28298888 PMC5331030

[B229] Sporns O. (2013). Structure and function of complex brain networks. Dialog. Clin. Neurosci. 15:247. 10.31887/DCNS.2013.15.3/osporns 24174898 PMC3811098

[B230] Steinert S. Friedrich O. (2020). Wired emotions: ethical issues of affective brain-computer interfaces. Sci. Eng. Ethics 26, 351–367. 10.1007/s11948-019-00087-2 30868377 PMC6978299

[B231] Sternson S. M. Roth B. L. (2014). Chemogenetic tools to interrogate brain functions. Annu. Rev. Neurosci. 37, 387–407. 10.1146/annurev-neuro-071013-014048 25002280

[B232] Stocco A. Prat C. S. Losey D. M. Cronin J. A. Wu J. Abernethy J. A.. (2015). Playing 20 questions with the mind: collaborative problem solving by humans using a brain-to-brain interface. PLoS ONE 10:e0137303. 10.1371/journal.pone.0137303 26398267 PMC4580467

[B233] Stoica A. (2012). “Multimind: multi-brain signal fusion to exceed the power of a single brain,” in 2012 Third International Conference on Emerging Security Technologies (Lisbon: IEEE), 94–98. 10.1109/EST.2012.47

[B234] Summerer L. Izzo D. Rossini L. (2009). Brain-machine interfaces for space applications-research, technological development, and opportunities. Int. Rev. Neurobiol. 86, 213–223. 10.1016/S0074-7742(09)86016-9 19608002

[B235] Taylor D. M. Tillery S. I. H. Schwartz A. B. (2002). Direct cortical control of 3d neuroprosthetic devices. Science 296, 1829–1832. 10.1126/science.1070291 12052948

[B236] Tidoni E. Abu-Alqumsan M. Leonardis D. Kapeller C. Fusco G. Guger C.. (2016). Local and remote cooperation with virtual and robotic agents: a p300 BCI study in healthy and people living with spinal cord injury. IEEE Trans. Neural Syst. Rehabil. Eng. 25, 1622–1632. 10.1109/TNSRE.2016.2626391 28026777

[B237] Tonin L. Bauer F. C. Millán J. d. R. (2019). The role of the control framework for continuous teleoperation of a brain-machine interface-driven mobile robot. IEEE Trans. Robot. 36, 78–91. 10.1109/TRO.2019.2943072

[B238] Tonin L. Millán J. d. R. (2020). Noninvasive brain-machine interfaces for robotic devices. Annu. Rev. Control Robot. Auton. Syst. 4. 10.1146/annurev-control-012720-093904

[B239] Toriyama H. Ushiba J. Ushiyama J. (2018). Subjective vividness of kinesthetic motor imagery is associated with the similarity in magnitude of sensorimotor event-related desynchronization between motor execution and motor imagery. Front. Hum. Neurosci. 12:295. 10.3389/fnhum.2018.00295 30108492 PMC6079198

[B240] Tremmel C. Herff C. Sato T. Rechowicz K. Yamani Y. Krusienski D. J. (2019). Estimating cognitive workload in an interactive virtual reality environment using EEG. Front. Hum. Neurosci. 13:401. 10.3389/fnhum.2019.00401 31803035 PMC6868478

[B241] Vaadia E. Birbaumer N. (2009). Grand challenges of brain computer interfaces in the years to come. Front. Neurosci. 3:15. 10.3389/neuro.01.015.2009 20228862 PMC2751651

[B242] van Vliet M. Robben A. Chumerin N. Manyakov N. V. Combaz A. Van Hulle M. M. (2012). “Designing a brain-computer interface controlled video-game using consumer grade EEG hardware,” in 2012 ISSNIP Biosignals and Biorobotics Conference: Biosignals and Robotics for Better and Safer Living (BRC) (Manaus: IEEE), 1–6. 10.1109/BRC.2012.6222186

[B243] Vansteensel M. J. Pels E. G. Bleichner M. G. Branco M. P. Denison T. Freudenburg Z. V.. (2016). Fully implanted brain-computer interface in a locked-in patient with als. N. Engl. J. Med. 375, 2060–2066. 10.1056/NEJMoa1608085 27959736 PMC5326682

[B244] Vasilyev A. Liburkina S. Yakovlev L. Perepelkina O. Kaplan A. (2017). Assessing motor imagery in brain-computer interface training: psychological and neurophysiological correlates. Neuropsychologia 97, 56–65. 10.1016/j.neuropsychologia.2017.02.005 28167121

[B245] Vidal J. J. (1973). Toward direct brain-computer communication. Annu. Rev. Biophys. Bioeng. 2, 157–180. 10.1146/annurev.bb.02.060173.001105 4583653

[B246] Vidaurre C. Blankertz B. (2010). Towards a cure for BCI illiteracy. Brain Topogr. 23, 194–198. 10.1007/s10548-009-0121-6 19946737 PMC2874052

[B247] Vourvopoulos A. Pardo O. M. Lefebvre S. Neureither M. Saldana D. Jahng E.. (2019). Effects of a brain-computer interface with virtual reality (VR) neurofeedback: a pilot study in chronic stroke patients. Front. Hum. Neurosci. 13:210. 10.3389/fnhum.2019.00210 31275126 PMC6593205

[B248] Vyas S. Even-Chen N. Stavisky S. D. Ryu S. I. Nuyujukian P. Shenoy K. V. (2018). Neural population dynamics underlying motor learning transfer. Neuron 97, 1177–1186. 10.1016/j.neuron.2018.01.040 29456026 PMC5843544

[B249] Wang H. Chang W. Zhang C. (2016a). Functional brain network and multichannel analysis for the p300-based brain computer interface system of lying detection. Expert Syst. Appl. 53, 117–128. 10.1016/j.eswa.2016.01.024

[B250] Wang H. McIntosh A. R. Kovacevic N. Karachalios M. Protzner A. B. (2016b). Age-related multiscale changes in brain signal variability in pre-task versus post-task resting-state EEG. J. Cogn. Neurosci. 28, 971–984. 10.1162/jocn_a_00947 26942319

[B251] Wang W. Collinger J. L. Perez M. A. Tyler-Kabara E. C. Cohen L. G. Birbaumer N.. (2010). Neural interface technology for rehabilitation: exploiting and promoting neuroplasticity. Phys. Med. Rehabil. Clin. 21, 157–178. 10.1016/j.pmr.2009.07.003 19951784 PMC2788507

[B252] Wardman D. L. Gandevia S. C. Colebatch J. G. (2014). Cerebral, subcortical, and cerebellar activation evoked by selective stimulation of muscle and cutaneous afferents: an fMRI study. Physiol. Rep. 2:e00270. 10.1002/phy2.270 24771687 PMC4001872

[B253] Waytowich N. R. Lawhern V. J. Bohannon A. W. Ball K. R. Lance B. J. (2016). Spectral transfer learning using information geometry for a user-independent brain-computer interface. Front. Neurosci. 10:430. 10.3389/fnins.2016.00430 27713685 PMC5032911

[B254] Wei C.-S. Wang Y.-T. Lin C.-T. Jung T.-P. (2018). Toward drowsiness detection using non-hair-bearing EEG-based brain-computer interfaces. IEEE Trans. Neural Syst. Rehabil. Eng. 26, 400–406. 10.1109/TNSRE.2018.2790359 29432111

[B255] Wronkiewicz M. Larson E. Lee A. K. (2015). Leveraging anatomical information to improve transfer learning in brain-computer interfaces. J. Neural Eng. 12:046027. 10.1088/1741-2560/12/4/046027 26169961 PMC4527978

[B256] Wu D. King J.-T. Chuang C.-H. Lin C.-T. Jung T.-P. (2017a). Spatial filtering for EEG-based regression problems in brain-computer interface (BCI). IEEE Trans. Fuzzy Syst. 26, 771–781. 10.1109/TFUZZ.2017.2688423

[B257] Wu D. Lance B. J. Lawhern V. J. Gordon S. Jung T.-P. Lin C.-T. (2017b). EEG-based user reaction time estimation using riemannian geometry features. IEEE Trans. Neural Syst. Rehabil. Eng. 25, 2157–2168. 10.1109/TNSRE.2017.2699784 28463203

[B258] Wu D. Lawhern V. J. Hairston W. D. Lance B. J. (2016). Switching EEG headsets made easy: reducing offline calibration effort using active weighted adaptation regularization. IEEE Trans. Neural Syst. Rehabil. Eng. 24, 1125–1137. 10.1109/TNSRE.2016.2544108 27008670

[B259] Wu D. Xu Y. Lu B. (2020). Transfer learning for EEG-based brain-computer interfaces: a review of progress made since 2016. IEEE Trans. Cogn. Dev. Syst. 10.1109/TCDS.2020.3007453

[B260] Yang W. Miller J. K. Carrillo-Reid L. Pnevmatikakis E. Paninski L. Yuste R.. (2016). Simultaneous multi-plane imaging of neural circuits. Neuron 89, 269–284. 10.1016/j.neuron.2015.12.012 26774159 PMC4724224

[B261] Yin E. Zeyl T. Saab R. Chau T. Hu D. Zhou Z. (2015). A hybrid brain-computer interface based on the fusion of p300 and SSVEP scores. IEEE Trans. Neural Syst. Rehabil. Eng. 23, 693–701. 10.1109/TNSRE.2015.2403270 25706721

[B262] Yin M. Borton D. A. Komar J. Agha N. Lu Y. Li H.. (2014). Wireless neurosensor for full-spectrum electrophysiology recordings during free behavior. Neuron 84, 1170–1182. 10.1016/j.neuron.2014.11.010 25482026

[B263] Yu Y. Liu Y. Jiang J. Yin E. Zhou Z. Hu D. (2018). An asynchronous control paradigm based on sequential motor imagery and its application in wheelchair navigation. IEEE Trans. Neural Syst. Rehabil. Eng. 26, 2367–2375. 10.1109/TNSRE.2018.2881215 30442610

[B264] Yuan W. Li Z. (2018). Brain teleoperation control of a nonholonomic mobile robot using quadrupole potential function. IEEE Trans. Cogn. Dev. Syst. 11, 527–538. 10.1109/TCDS.2018.2869903

[B265] Zander T. O. Kothe C. Welke S. Rötting M. (2009). “Utilizing secondary input from passive brain-computer interfaces for enhancing human-machine interaction,” in International Conference on Foundations of Augmented Cognition (Las Vegas, NV: Springer), 759–771. 10.1007/978-3-642-02812-0_86

[B266] Zhang R. Xu P. Chen R. Li F. Guo L. Li P.. (2015). Predicting inter-session performance of SMR-based brain-computer interface using the spectral entropy of resting-state EEG. Brain Topogr. 28, 680–690. 10.1007/s10548-015-0429-3 25788102

[B267] Zhang W. Wu D. (2020). Manifold embedded knowledge transfer for brain-computer interfaces. IEEE Trans. Neural Syst. Rehabil. Eng. 28, 1117–1127. 10.1109/TNSRE.2020.2985996 32286993

[B268] Zhao X. Chu Y. Han J. Zhang Z. (2016). SSVEP-based brain-computer interface controlled functional electrical stimulation system for upper extremity rehabilitation. IEEE Trans. Syst. Man Cybernet. Syst. 46, 947–956. 10.1109/TSMC.2016.2523762

[B269] Zuo C. Jin J. Yin E. Saab R. Miao Y. Wang X.. (2020). Novel hybrid brain-computer interface system based on motor imagery and p300. Cogn. Neurodyn. 14, 253–265. 10.1007/s11571-019-09560-x 32226566 PMC7090135

